# Highly attenuated dendritic propagation of isolated synaptic potentials in vivo

**DOI:** 10.1126/sciadv.adz4123

**Published:** 2026-08-14

**Authors:** Victor Hugo Cornejo, Boris Bouazza-Arostegui, Tzitzitlini Alejandre-Garcia, Yukun Hao, Sungmoo Lee, Michael Z. Lin, Rafael Yuste

**Affiliations:** ^1^ Neurotechnology Center, Department of Biological Sciences, Columbia University, New York, NY 10027, USA.; ^2^ Department of Bioengineering, Stanford University, Stanford, CA 94305, USA.

## Abstract

The integration of synaptic inputs is a fundamental function of neurons. In the traditional model, excitatory inputs are summed at the soma to generate action potentials. However, how synaptic inputs are integrated by dendrites in vivo remains poorly explored. We used intravital two-photon dendritic imaging with a genetically encoded voltage indicator (accelerated sensor of action potentials 5) together with somatic whole-cell patch clamp recordings to investigate how synaptic depolarizations are transferred to the soma in pyramidal neurons of the mouse somatosensory cortex. We studied the integration of synaptic inputs under spontaneous and sensory-evoked conditions, as well as following electrical and optogenetic stimulation. In all cases, while multiple inputs evoked measurable depolarizations in the cell body, isolated synaptic potentials were strongly attenuated. Our results suggest that isolated synaptic inputs have a minimal contribution to somatic depolarization, whereas coincident inputs within short temporal windows are more effective, indicating a regime of dendritic integration that favors coincident or clustered neuronal activity in cortical networks.

## INTRODUCTION

Neurons integrate and transform synaptic inputs to generate action potentials (APs) (*1*, *2*). Decades of neurophysiological and biophysical research have demonstrated the functional operation of excitatory and inhibitory postsynaptic potentials (EPSPs and IPSPs, respectively) (*3*–*7*) and shown how their interaction leads to the generation of APs, typically in the soma or the axonal initial segment (*8*–*10*). Notably, mammalian cortical neurons of layer 2/3 typically receive on the order of 8000 excitatory synaptic inputs (*11*), positioned in different dendritic regions that allow diverse integration modalities (*3*, *12*). From the site of generation at dendritic spines, excitatory dendritic membrane potentials undergo transformations on the way to the cell body. Synaptic inputs are attenuated along the dendritic arbor because of their passive membrane properties and the morphology of the dendritic arborization, according to cable theory (*13*–*19*). In addition, dendrites exhibit active electrical properties conferred principally by voltage-gated channels expressed in the dendritic tree (*20*–*23*). This property endows dendrites with the capacity not only to boost distal signals but also to support clustering and the detection of multiple synchronous synaptic inputs (*9*, *24*–*28*). The collection of active and passive properties of dendrites has been studied profusely in numerical simulations and in vitro experiments (*29*), but they may not fully capture the biological complexity of the native tissue environment. Neurons receive much larger amounts of synaptic inputs in vivo. This constant barrage of inputs substantially alters the physiological properties in the native context, along with inhibitory circuits important for the efficacy of synaptic transmission in the brain (*3*, *23*, *30*–*33*).

Pioneering in vivo studies on dendritic integration in pyramidal neurons have characterized various signals across different dendritic compartments in animals, spanning multiple cortical areas, using patch clamp electrophysiology and calcium imaging techniques (*34*–*50*). Despite substantial technical and conceptual advances, these studies may have only captured a portion of the diverse signals driven by synapses and dendrites intravitally. Therefore, full characterization of the voltage signals arising from synaptic inputs and the transformation mechanisms is essential to understand the intricate process of input integration in vivo (*51*–*56*).

Recent advancements in imaging techniques for assessing neuronal membrane voltage in the brain highlight the potential to introduce a new layer of complexity to intact tissue connectivity and biological context (*57*–*60*). In particular, imaging of postsynaptic potentials in cortical pyramidal neurons in vivo using genetically encoded voltage indicators (GEVIs) has revealed that the activation of dendritic spines is local, suggesting that synaptic inputs may not always reach the soma (*61*). To investigate this phenomenon further, we used a GEVI called “accelerated sensor of APs 5” (ASAP5), capable of measuring unitary synaptic events (*62*), to image membrane potential in basal dendrites with simultaneous somatic whole-cell patch clamp recordings in anesthetized mice, during spontaneous or sensory-evoked activity, or by direct synaptic activation with electrical stimulation or optogenetics. Our findings indicate that a large fraction of dendritic depolarizations generated by isolated synaptic events, defined here as spatially and temporally restricted synaptic events without detectable coincident depolarization in neighboring dendritic regions, are highly attenuated and become undetectable at the soma. In contrast to these isolated events, synaptic events associated with coincident activity produce measurable somatic depolarizations. These results indicate that, under the experimental conditions tested, dendritic integration in layer 2/3 pyramidal neurons operates in a regime in which spatially and temporally isolated inputs are strongly attenuated, whereas coincident inputs are more effective at influencing somatic membrane potential.

## RESULTS

### In vivo calibration of GEVI signals with somatic patch clamp

**Fig. 1.**
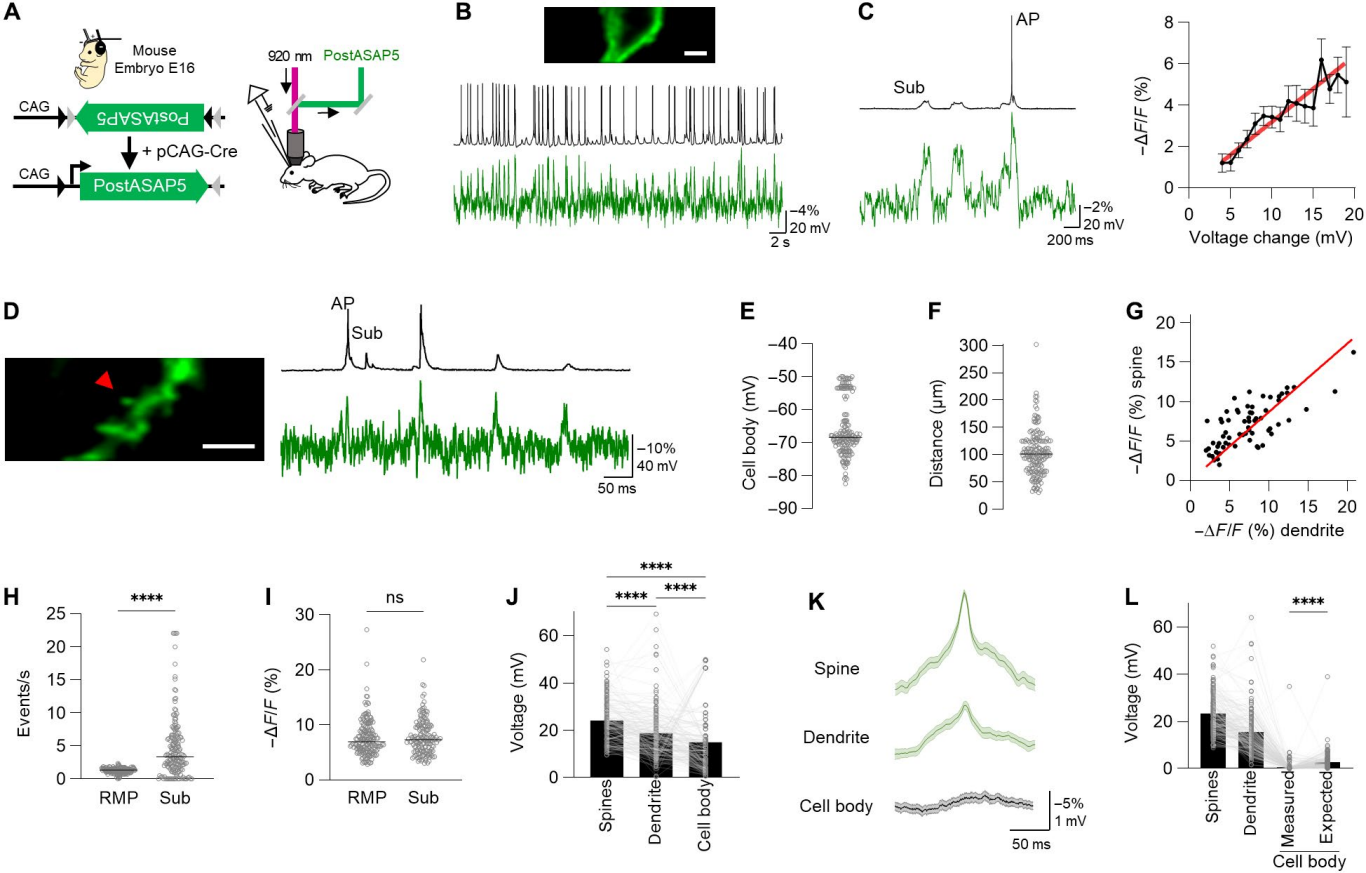
Spines and somatic responses during spontaneous activity. (**A**) The pCAG-postASAP5-flex vector and pCAG-Cre were coelectroporated into the prospective somatosensory cortex. Mice were implanted with a cranial window for simultaneous two-photon imaging and whole-cell recordings in vivo. (**B**) Simultaneous whole-cell recording and two-photon imaging. Top: A cell body of a neuron expressing postASAP5. Scale bar, 5 μm. Bottom: Simultaneous whole-cell voltage trace (black) and optical signal (green). (**C**) Example traces showing subthreshold (Sub) and AP events. Right: Correlation between postASAP5 fluorescence and subthreshold electrical amplitudes (means ± SD; *n* = 435 subthreshold events, 28 cells, 16 animals; linear regression: *y* = 0.32*x*, *R*^2^ = 0.87, *P* = 0.0001). (**D**) Dendritic segment and spines expressing postASAP5. Scale bar, 5 μm. Right: Corresponding somatic recording (black) and optical trace from the indicated spine (red arrowhead, green trace). (**E**) Resting membrane potential (RMP) of analyzed spines (*n* = 172 spines). (**F**) Distance from spines to the soma (*n* = 172 spines). (**G**) Peak fluorescence during APs in spines and dendrites (*n* = 70 spines). (**H**) Frequency of spine events detected during RMP and subthreshold epochs in the soma (*n* = 172 spines, ****P < 0.0001). (**I**) Fluorescence changes in spine events detected during RMP and subthreshold epochs in the soma (*n* = 172 spines). (**J**) Comparison of voltages estimated in spines and dendrites and the electrical voltage recorded in the soma during subthreshold events (*n* = 152 spines; Kruskal-Wallis test, multiple comparisons, ****P < 0.0001). (**K**) Representative averaged traces (20 events, means ± SD) of one spine and dendrite (green) with time-locked somatic voltage recordings (black). (**L**) Correlation between voltages estimated in spines and dendrites and the electrical voltage recorded in the soma during RMP epochs. Comparison between measured and expected somatic voltages (*n* = 169 spines; Mann-Whitney test, ****P < 0.0001).

To image membrane potential in vivo, we used ASAP5, a recently developed GEVI with fast kinetics and capable of capturing synaptic events in vitro (*62*). We modified the construct by incorporating the PSD95.FingR nanobody (*63*) to enhance its localization to dendrites and spines (*61*). To express it in pyramidal neurons, we cloned this modified construct, hereafter referred to as “postASAP5,” under the control of the CAG promoter and a Cre-loxP recombination system (Fig. 1A). Using in utero electroporation in mice, we achieved strong yet sparse expression of postASAP5 in pyramidal cells, enabling clear visualization of dendritic morphologies, including spines. We imaged layer 2/3 postASAP5-expressing pyramidal neurons in primary somatosensory cortex in vivo, using a modified two-photon microscope operating at 350-Hz frame rate, and performed simultaneous whole-cell patch clamp recordings from their cell bodies (*n* = 28 neurons, 16 animals; Fig. 1B). To calibrate the fluorescent response of postASAP5 to synaptic potentials, we monitored subthreshold depolarization under light isoflurane anesthesia (*64*–*67*) and quantified their peak amplitudes along with time-locked peak fluorescent responses in the soma (see Materials and Methods and Fig. 1C). During spontaneous activity, we observed a continuous barrage of fluorescence somatic responses that corresponded to subthreshold depolarizations measured with the patch pipette, with a linear response over a 20-mV range from resting potentials (*y* = 0.32*x*, *R*^2^ = 0.87, and *P* = 0.0001). These optical or electrical depolarizing events were blocked after perfusion of natural cerebrospinal fluid (CSF) with tetrodotoxin (TTX) at the voltage imaging site, confirming their physiological origin (fig. S1, A and B). These experiments established that postASAP5 reports subthreshold depolarizations in vivo with linearity and physiological specificity. We note, however, that this calibration is valid only within the linear operating range, as signals outside this range may lead to quantitative underestimation.

### Spontaneous spine events show minimal somatic impact

We next conducted voltage imaging during ongoing spontaneous activity in basal dendrites, focusing on both dendritic shafts and spines, while simultaneously performing somatic electrical recordings (*n* = 22 neurons, 19 animals; Fig. 1D). Subthreshold depolarizations measured at the soma frequently displayed depolarizing signals in spines, with fluorescence levels often comparable to those observed during AP firing (Fig. 1D). For this experiment, we analyzed 172 spines, along with somatic patch clamp recordings over a 30-s period, with a resting membrane potential (RMP) of −65.80 ± 8.46 mV (means ± SD) (Fig. 1E). These spines were located at 102.7 ± 41.1 μm (means ± SD) away from the cell body (Fig. 1F). Consistent with previous reports (*8*, *9*, *34*–*36*, *68*–*70*), individual APs at the soma generated depolarizations in both dendrites and spines, with an estimated activation probability of 85.4%, corresponding to the proportion of events that exceeded a signal-to-noise ratio (SNR) of >2 (fig. S2B). We also observed a moderate negative correlation between AP amplitude and distance from the soma, consistent with cable theory predictions of amplitude decay along the dendritic path (fig. S3A). At the local level, AP depolarizations propagated from dendrites into spines, with a linear correlation between signals in these compartments [spine = 6.92 ± 2.82 − Δ*F*/*F*% and dendrite = 7.25 ± 3.75 − Δ*F*/*F*% (means ± SD); *y* = 0.87*x*, *R*^2^ = 0.53, and *P* < 0.0001; Fig. 1G]. These results confirm backpropagation of APs into spines and dendrites, with a strong correlation of optical signals across compartments.

We then investigated whether spines were also active during periods of subthreshold somatic depolarization or when the soma remained at RMP. To differentiate significant depolarization signals in the spines from shot noise, we applied a stringent automatic detection algorithm using an SNR threshold of >2 for at least two consecutive points in the signals, while preserving the signal shape. Because the sensitivity of current GEVI does not allow reliable detection of the smallest synaptic events in vivo, we relied on trial-averaged signals to improve SNR; for each spine, detected events within epochs of subthreshold activity or RMP were averaged across the recording to enable quantification of depolarizations (fig. S2C). Across all spines, detected depolarization events occurred at an average frequency of 1.49 ± 0.36 events/s (means ± SD) during the recording period. These spine events were more frequent during subthreshold somatic depolarizations, as compared to RMP epochs [RMP = 1.27 ± 0.39 events/s and subthreshold = 4.59 ± 4.54 events/s (means ± SD); Mann-Whitney test, *P* < 0.0001; Fig. 1H]. The amplitude of the spine events detected during subthreshold somatic events was similar to those during RMP [RMP = 7.65 ± 3.30 − Δ*F*/*F*% and subthreshold = 7.85 ± 3.18 − Δ*F*/*F*% (means ± SD); Mann-Whitney test, *P* = 0.467; Fig. 1I]. Estimated voltages were calculated on the basis of the linear regression in Fig. 1C, allowing the transformation of fluorescence changes into voltage values for comparison across compartments. For detected fluorescent spine events, we report averages aligned to the detected events and measured peak voltages in dendritic shafts and the soma using time-locked signals. During somatic subthreshold potentials, spine voltages were slightly higher than those in dendrites and the soma, as expected from integration of multiple synaptic inputs [estimated spine voltage = 24.05 ± 9.02 mV, estimated dendritic voltage = 18.81 ± 11.16 mV, and measured cell body = 14.97 ± 12.92 mV (means ± SD); Fig. 1J].

We further focused on spine events occurring at RMP (Fig. 1K). Notably, most spine optical events (80.1%) showed minimal or no detectable corresponding somatic electrical signals (SNR < 2), even when significant signals were present in spines [estimated spine voltage = 23.31 ± 8.64 mV, estimated dendritic voltage = 15.37 ± 10.30 mV, and measured cell body = 0.579 ± 2.907 mV (means ± SD); Fig. 1L]. Neither the amplitude nor the attenuation of spine or dendrite events correlated with spine-to-soma distance (fig. S3B). Using an exponential decay function, the measured distances between spines and the soma (Fig. 1F), and a previously published electrotonic effective length constant for pyramidal cortical neurons (*12*, *32*, *62*, *71*), we calculated the expected somatic voltage decay using a passive cable model. Unexpectedly, the observed somatic voltage was significantly lower than the predicted value [expected cell body = 2.66 ± 3.58 mV (means ± SD); Mann-Whitney test, *P* < 0.0001; Fig. 1L]. These findings indicate that many synaptic events remain confined to spines and dendrites, with minimal somatic impact, diverging from passive cable numerical predictions.

### Sensory-evoked inputs exhibit strong dendritic attenuation

**Fig. 2.**
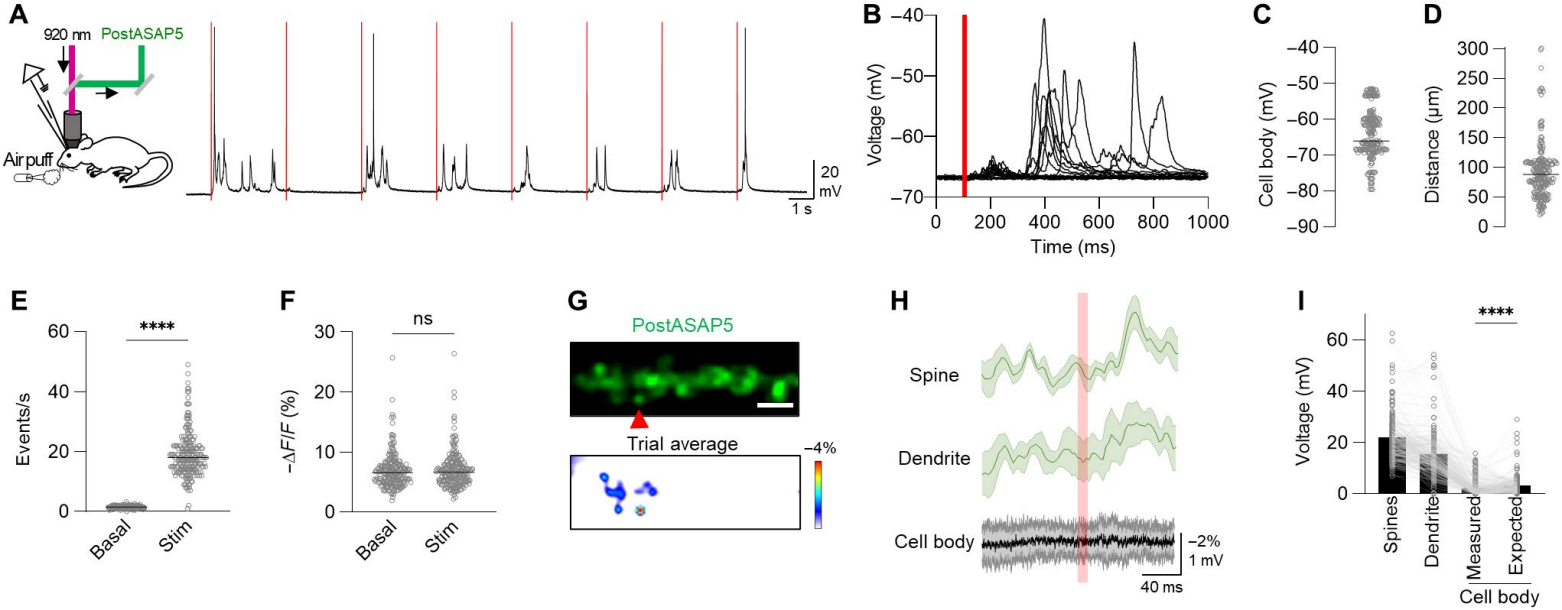
Spines and somatic responses during sensory stimulation. (**A**) Example of a whole-cell patch clamp recording in current-clamp mode from a pyramidal neuron in the somatosensory cortex during a 10-ms air puff to the whiskers (red vertical line). (**B**) Membrane voltage recording following whisker stimulation (Stim; 10 trials). Air puffs were locked at 100 ms (red line). (**C**) RMP of analyzed spines (*n* = 220 spines). (**D**) Distance from spines to the soma (*n* = 220 spines). (**E**) Frequency of spine events detected during basal activity and after stimulation (1 s after air puff, *n* = 220 spines, ****P < 0.0001). (**F**) Fluorescence changes in spine events detected during basal activity and after stimulation (*n* = 220 spines). (**G**) Representative voltage imaging of a dendritic segment and spines. Image averaged over 10 trials (100 ms after stimulus onset). Signals with an SNR of ≥2 are displayed. Scale bar, 5 μm. (**H**) Trial-averaged signals following whisker stimulation (10 trials; means ± SD). Voltage imaging fluorescence change in a ROI drawn around the spine [red arrowhead in (G)] and its adjacent dendritic segment (green traces). The bottom trace (black) shows the average electrical signal recorded in the soma. (**I**) Correlation between voltages estimated in spines and dendrites and the electrical voltage recorded in the soma after sensory stimulation. Comparison between measured and expected somatic voltages (*n* = 219 spines; Mann-Whitney test, ****P < 0.0001).

We next explored whether a similar attenuation of synaptic events could also occur during sensory-evoked responses. To test this, we investigated the relationship between spine activation and somatic depolarization in response to unilateral sensory stimulation of the mouse vibrissae. Using short air puffs (10 ms; 10 repetitions at 3-s intervals), we stimulated contralateral whiskers while simultaneously imaging spine and dendritic potentials along with electrical membrane potential from the soma (*n* = 10 neurons, eight animals; Fig. 2A). Following sensory stimulation, we observed somatic depolarizations comprising a combination of subthreshold events and AP firing (Fig. 2A), consistent with previous observations in the barrel cortex (*72*–*74*). To better distinguish the effects of sensory stimuli on the neuron from spontaneous activity, we increased the anesthetic concentration to reduce ongoing activity barrages (fig. S4, A and B), since ongoing activity has been shown to inhibit sensory responses evoked by whisker deflection (*72*). Closer examination of the somatic membrane potentials during the first second after whisker stimulation revealed an initial somatic response characterized by low-amplitude, desynchronized events lasting approximately 200 ms, followed by a subsequent increase in activity with higher-amplitude depolarizations (Fig. 2B).

As before, we conducted patch clamp recordings in the cell body [RMP = −64.20 ± 6.99 mV (means ± SD); Fig. 2C] and performed voltage imaging in 220 spines located 92.4 ± 45.9 μm (means ± SD) away from the soma (Fig. 2D). Spine depolarization events were extracted, classified, and averaged to improve SNR (fig. S2D), on the basis of their timing relative to sensory stimulation, with sensory events defined as occurring within 1 s after the air puff, compared to the entire recording duration. We observed a significant increase in spine events following sensory stimulation [basal = 1.47 ± 0.41 events/s, stimulation = 19.10 ± 7.50 events/s (means ± SD); Mann-Whitney test, *P* < 0.0001; Fig. 2E]. However, the amplitude of events occurring after stimulation was not significantly different from those during the basal period [basal = 6.89 ± 2.86 − Δ*F*/*F*% and stimulation = 7.02 ± 2.98 − Δ*F*/*F*% (means ± SD); Mann-Whitney test, *P* = 0.601; Fig. 2F].

To examine whether depolarizing responses from the spines reached the soma, we measured the time-locked peak fluorescence in spines and neighboring dendrites, while recording with patch clamp the voltage in the cell body. Although spine and dendritic depolarization was significant, no corresponding activation was observed in the soma (Fig. 2, G and H). Forty-eight percent of these somatic electrical responses during sensory stimulation were indistinguishable from noise. As previously, estimating the electrical responses in spines and dendrites using voltage imaging data and comparing these with somatic voltages, we found a significant decay from spines to dendrites and finally to the soma [estimated spine voltage = 21.96 ± 9.33 mV, estimated dendrite voltage = 15.45 ± 11.79 mV, and measured cell body = 2.14 ± 3.26 mV (means ± SD); Kruskal-Wallis test, *P* < 0.0001; Fig. 2I]. Moreover, we found only a weak correlation between the amplitude of spine or dendritic depolarizations and spine-to-soma distance and essentially no correlation between distance and the degree of signal attenuation (fig. S3C). Applying a passive cable model to our measured spine-to-soma distances, we estimated the expected voltage in the soma, finding that it was significantly higher than the actual measured value [expected cell body = 3.17 ± 3.67 mV (means ± SD); Mann-Whitney test, *P* < 0.0001; Fig. 2I]. These results suggest that in vivo sensory activation generates a broad repertoire of synaptic and dendritic events, reflected in somatic integration. However, individual spines generate responses that are attenuated, as they propagate toward the soma.

### Multiple synaptic inputs drive measurable somatic depolarization

**Fig. 3.**
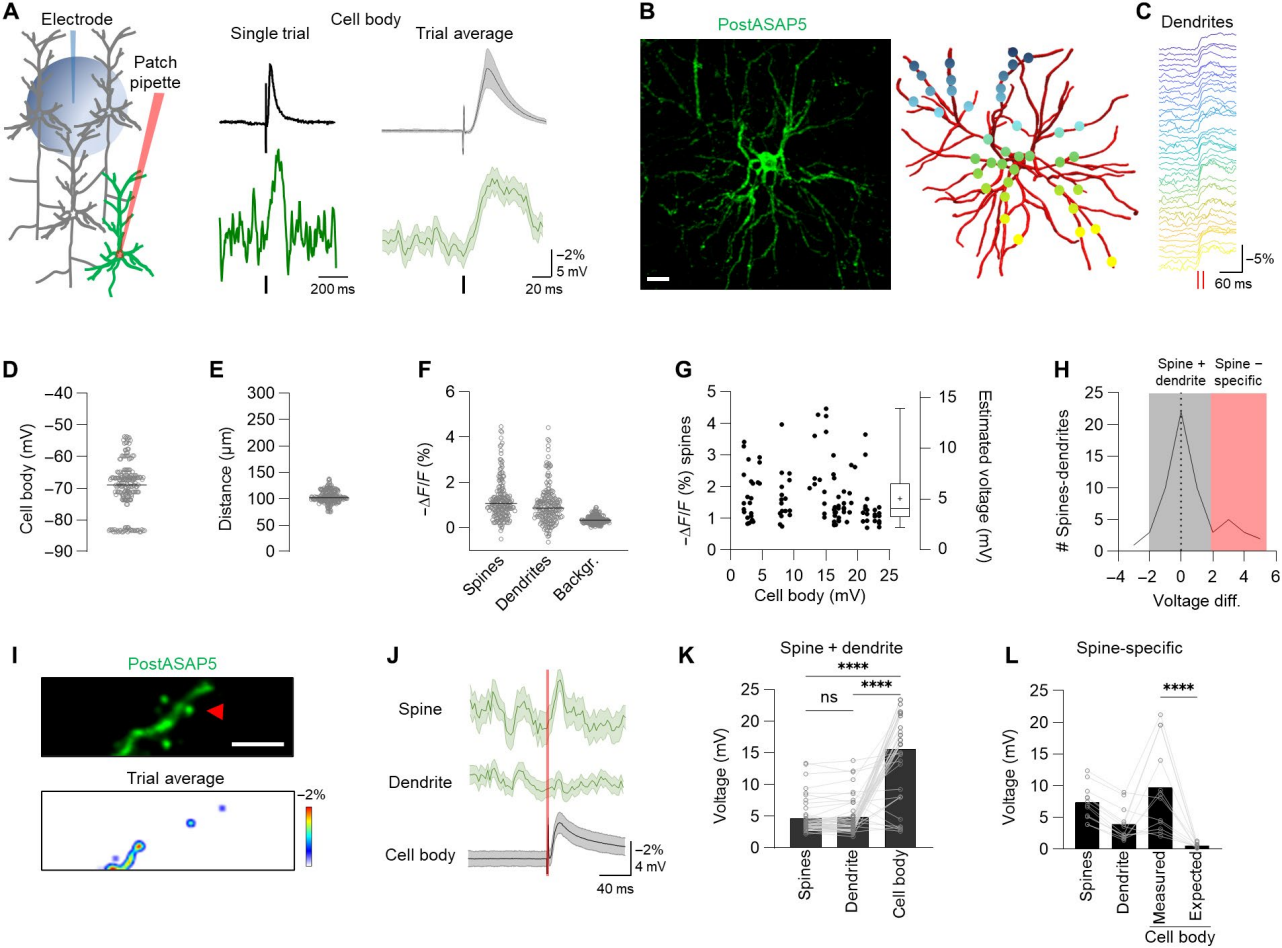
Spines and somatic responses during electrical stimulation. (**A**) Electrical stimulation was applied to the somatosensory cortex neuropil (∼1 mm from the postsynaptic recording site). Right: Somatic responses measured by whole-cell recording and voltage imaging (30-trial average, means ± SD). (**B**) Three-dimensional reconstruction of a postASAP5-expressing pyramidal neuron with dendritic skeletonization and stimulation site indicated 1 mm away. Scale bar, 20 μm. (**C**) Mapping of dendritic voltage imaging from the neuron shown in (B) following paired-pulse stimulation (25-ms interval). (**D**) RMP of analyzed spines (*n* = 197 spines). (**E**) Distance from spines to the soma (*n* = 197 spines). (**F**) Fluorescence changes detected within 50 ms after stimulation for spines, adjacent dendrites, and background (*n* = 197 spines). (**G**) Relationship between spine fluorescence and somatic voltage responses. Right: Box plot of estimated spine voltages (median line; mean indicated by “+”; *n* = 121 spines; Mann-Whitney, *P* < 0.0001; *R*^2^ = 0.02, *P* = 0.12). (**H**) Distribution of voltage differences between spine and dendrite (*n* = 59 spines). Gray, spine + dendrite events (similar amplitudes); red, spine-specific events (enhanced spine depolarization). (**I**) Example voltage imaging of dendritic segment with spines. Image averaged over 30 trials (50 ms after stimulus onset). Signals with an SNR of ≥2 are displayed. Scale bar, 5 μm. (**J**) Trial-averaged signals (red line, 30 trials; means ± SD). Voltage imaging fluorescence change in a spine [red arrowhead in (I)] and its adjacent dendritic segment (green trace). Bottom trace: Averaged electrical signal recorded in the soma (black trace). (**K**) Correlation between voltages estimated in spines and dendrites and the electrical voltage recorded after stimulation for spine + dendrite events (*n* = 47; Kruskal-Wallis test; ****P < 0.0001). (**L**) Same as in (K) for spine-specific events (*n* = 12 spines; Kruskal-Wallis test; ****P < 0.0001).

Our results indicated that a subset of synaptic events during spontaneous or sensory-driven activity exerted a negligible effect on the soma, whereas another subset, likely reflecting the convergence of multiple inputs, was capable of producing detectable depolarization. To further interrogate this phenomenon, we next sought to recapitulate it under conditions in which subthreshold events could be experimentally triggered through controlled synaptic activation. To minimize ongoing activity, we increased the anesthetic concentration and used this setting for all subsequent experiments to better isolate synaptic responses. We then applied extracellular stimulation in the neighboring neuropil, imaging spine, and dendritic voltage dynamics while simultaneously recording somatic depolarization with patch clamp (*n* = 10 neurons, seven animals; Fig. 3A). To confirm that remote electrical stimulation effectively activated axons at the site of imaging, we performed calcium imaging with an axon-specific calcium indicator, which demonstrated presynaptic activation located within 1 mm of the postsynaptic recording site (fig. S5, A and B). Stimulation parameters were calibrated to elicit detectable and reliable postsynaptic voltage responses (fig. S5C). In addition, stimulation protocols were validated through calcium imaging of postsynaptic spines, confirming direct synaptic activation (fig. S5, D and E).

We observed robust subthreshold depolarizations in the cell body during electrical stimulation, with trial-to-trial reliability confirmed by patch clamp recordings and somatic voltage imaging (Fig. 3A). Volumetric imaging of the dendritic tree revealed that paired-pulse presynaptic stimulation elicited widespread depolarizations throughout the dendritic arbor (Fig. 3, B and C). We then applied single-pulse electrical stimulation, imaging a total of 197 spines along with somatic patch clamp measurements across 30 trials. The RMP was −69.70 ± 7.90 mV (means ± SD) (Fig. 3D). Spines analyzed in this experiment were located at an average distance of 104.1 ± 11.7 μm (means ± SD) from the soma (Fig. 3E). Fluorescence changes of the voltage sensor were automatically detected within a 50-ms window after presynaptic stimulation for each region of interest (ROI), including spines, adjacent dendritic segments, and background noise [spines = 1.23 ± 0.98 Δ*F*/*F*%, dendrites = 0.98 ± 0.88 Δ*F*/*F*%, and background = 0.35 ± 0.18 Δ*F*/*F*% (means ± SD); Fig. 3F]. To ensure rigorous selection of events for subsequent analysis, we applied two criteria: (i) a threshold of 2 SDs above background noise and (ii) an SNR of >2 for trial-averaged signals (fig. S2E). Using these criteria, significant fluorescent spine voltage signals were plotted against the corresponding somatic electrical recording (Fig. 3G). On average, individual estimated spine voltages were significantly lower than and uncorrelated with recorded somatic voltages, consistent with summation across multiple spines [estimated spine voltage = 5.04 ± 2.68 mV and measured cell body = 13.27 ± 6.96 mV (means ± SD); *R*^2^ = 0.02, *P* = 0.12; Fig. 3G].

Pure voltage imaging cannot unambiguously separate synaptic activation of an individual spine from other potential sources of local depolarization. To address this, we further analyzed adjacent dendritic signals using the same SNR criteria applied to spines, identifying that 48.8% of events displayed significant and concomitant signals in both spines and dendrites. For this subset, we subtracted dendritic voltages from spine voltages (Fig. 3H). On the basis of this distribution, we categorized events as spine-specific, representing 20.3% of cases with higher depolarization in spines, or as spine + dendrite, characterized by statistically equivalent depolarizations occurring simultaneously in spines and dendrites. Accordingly, detailed trial-averaged imaging analysis revealed that depolarization often remained confined to dendritic spines but could also spread into adjacent dendritic segments (Fig. 3I) and was consistently associated with concomitant somatic depolarization (Fig. 3J). Further analyses suggested that spine + dendrite events had lower amplitudes compared with somatic responses, likely reflecting summation and spread across the dendritic tree, and activation of dendrites in regimes where contiguous spines were depolarized [estimated spine voltage = 4.71 ± 2.69 mV, estimated dendritic voltage = 4.86 ± 2.81 mV, and measured cell body = 15.63 ± 6.34 mV (means ± SD); Fig. 3K]. When analyzed separately, most somatic responses were also larger than spine-specific responses (Fig. 3L), consistent with integration across the dendritic tree [estimated spine voltage = 7.37 ± 2.70 mV, estimated dendritic voltage = 3.92 ± 2.65 mV, and measured cell body = 9.74 ± 7.12 mV (means ± SD); Fig. 3L]. Together, these findings demonstrate that multiple synaptic activations and widespread dendritic depolarization generate depolarizations in both spines and the soma. Thus, somatic depolarization is primarily driven by multiple, temporally coincident synaptic inputs.

### Optogenetically evoked isolated inputs are strongly attenuated at the soma

**Fig. 4.**
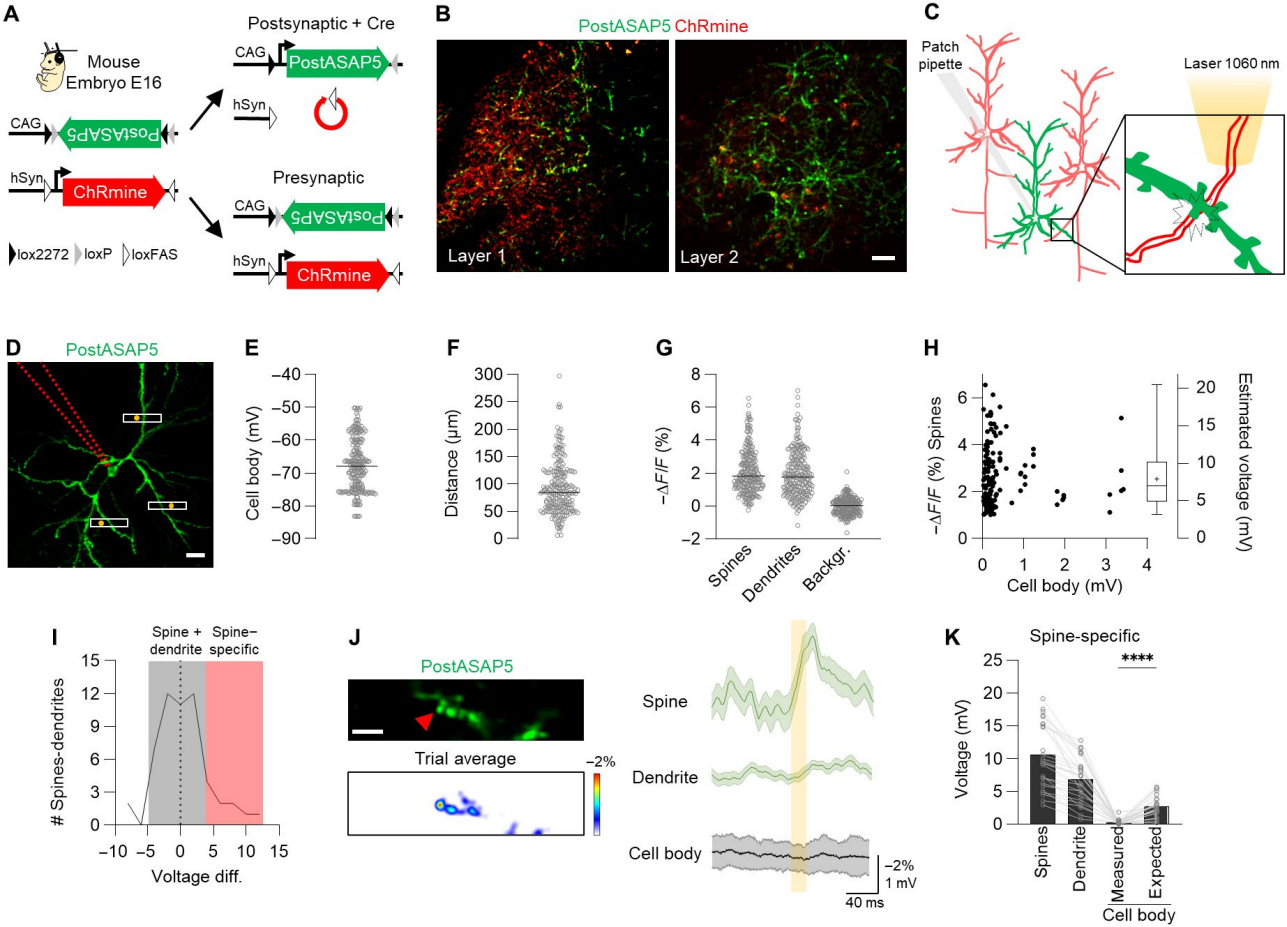
Spines and somatic responses during optogenetic stimulation. (**A**) Orthogonal expression of ChRmine in presynaptic neurons and postASAP5 in postsynaptic neurons. (**B**) Two-photon images showing ChRmine-mScarlet and postASAP5 expression in layers 1 and 2 (50 to 200 μm from pia). Scale bar, 50 μm. (**C**) Local stimulation applied near a dendritic segment. (**D**) Three-dimensional projection of a postASAP5-expressing pyramidal neuron showing optical stimulation sites (orange circles), ROIs for voltage imaging (white rectangles), and patch pipette (red dotted line). Scale bar, 25 μm. (**E**) RMP of analyzed spines (*n* = 223 spines). (**F**) Distance from spines to the soma (*n* = 223 spines). (**G**) Fluorescence peaks automatically detected within 100 ms after stimulation for spines and corresponding dendritic segments (*n* = 223); background peaks are measured from matched pixels. (**H**) Relationship between spine fluorescence and somatic voltage responses. Right: Box plot of estimated spine voltages (median line; mean indicated by “+”; *n* = 172 spines; Mann-Whitney, *P* < 0.0001). (**I**) Distribution of voltage differences between spine and dendrite (*n* = 54 spines). Gray, spine + dendrite events (similar amplitudes); red, spine-specific events (enhanced spine depolarization). (**J**) Representative voltage imaging of a dendritic segment and its spines. Bottom: Image average over 35 trials (50 ms poststimulus). Only signals with an SNR of ≥2 are displayed. Scale bar, 5 μm. Right: Trial-averaged voltage signals after optogenetic stimulation (yellow line) showing fluorescence changes in the ROI of the spine (red arrowhead in D) and its adjacent dendritic segment (green traces). The bottom black trace shows the average electrical signal from somatic patch clamp recordings (means ± SD). (**K**) Correlation between voltages estimated in spines and dendrites and the electrical voltage recorded in the soma after optogenetic stimulation. Comparison between measured and expected somatic voltages (*n* = 29 spines; Mann-Whitney test, ****P < 0.0001).

Previous experiments showed that synaptic activation, whether during spontaneous activity, sensory input, or electrical stimulation, produced a range of local depolarizations in spines and adjacent dendritic shafts, with varying degrees of somatic depolarization, or, in some cases, none at all. In all scenarios, numerous synapses distributed across the dendritic tree were likely activated together, leading to somatic depolarizations that resulted from the summation of many synaptic inputs. To specifically investigate the response to focal intracortical activation, generated by local synaptic inputs, we aimed to stimulate individual synapses (or a small number of them) using optogenetic activation of local axons (*75*–*78*), while simultaneously performing voltage imaging of the spines and dendrites, and somatic whole-cell patch clamp recordings (*n* = 27 neurons, 20 animals). Together with postASAP5, we expressed the optically orthogonal opsin ChRmine (*79*) with a mutually exclusive genetic system (*80*), labeling postsynaptic neurons with the voltage sensor and presynaptic cells with the red-shifted opsin (Fig. 4, A and B). Once expressed in the mouse cortex, photoactivating these neurons with a field light-emitting diode generated a postsynaptic response, while ensuring that opsin expression was excluded in the cells expressing the voltage sensor (fig. S6, A to C). Subsequently, we performed two-photon optogenetic stimulation with a 1060-nm laser and used a circular perisomatic spiral stimulation (20 μm in diameter), generating measurable postsynaptic responses at 50- and 20-ms stimulation in some spots (fig. S6, D to G). We also patch clamped a neuron expressing ChRmine and stimulated a proximal portion of the axon to determine the optimal timing for inducing presynaptic firing, ruling out a significant cross-talk effect from the imaging laser (fig. S7, A and B). Our experimental approach consisted of two-photon optogenetic recruitment of axons to activate only a small field of view near the imaged dendrite (Fig. 4C). We initially tested the viability of this strategy by stimulating axons in proximity to a postsynaptic neuron expressing GCaMP8s. A 50-ms stimulation elicited detectable calcium events in spines and dendrites (fig. S7, C and D).

We proceeded to activate only a small spot (10-μm spiral) for 20 ms near the imaged dendrites labeled with postASAP5, thereby restricting the number of activated axons and corresponding activated spines and dendrite (Fig. 4D). A total of 223 spines were measured, along with somatic patch clamp [35 trials, RMP = −65.50 ± 7.95 mV (means ± SD); Fig. 4E]. Voltage imaging data were analyzed following optogenetic presynaptic stimulation from spines located 92.5 ± 47.9 μm (means ± SD) away from the cell body (Fig. 4F). Fluorescence change peaks of the voltage sensor were automatically detected 50 ms after stimulation for each ROI, including spines, adjacent dendritic segments, and background noise [spines = 2.11 ± 1.34 − Δ*F*/*F*%, dendrite = 1.98 ± 1.40 − Δ*F*/*F*%, and background = −0.008 ± 0.51 − Δ*F*/*F*% (means ± SD); Fig. 4G]. To determine the cutoff for spine signals for further processing, we used the same SNR criteria as before for electrical-triggered events (fig. S2F). Then, we correlated the amplitudes of the somatic depolarization and the measured peak fluorescent responses time-locked to stimulation at the spines after optogenetics, revealing the low amplitude of most of somatic depolarization and low correlation between both signals [estimated spine voltage = 7.91 ± 3.82 mV and measured cell body = 0.37 ± 0.67 mV (means ± SD); *r* = 0.0037, *P* = 0.96; Fig. 4H]. We hypothesized that a portion of the somatic activity could also reflect dendritic depolarizations initiated by the convergence of multiple spine inputs, rather than solely by single-spine events. To test this, we identified significant dendritic depolarization peaks (159 spines) using the same SNR cutoff criteria. Within this dataset, 64.8% of spine signals occurred either before or concurrently with dendritic signals, which we attributed to synaptic activation–induced depolarization. For the purposes of this analysis, we operationally defined isolated synaptic potentials as events detected in spines that were not accompanied by coincident depolarization in adjacent dendritic regions or at the soma within a 50-ms temporal window. We therefore focused on a subset of spines that exhibited no delay in their responses (34.0%). Within this subset, we then plotted the difference between the estimated voltages in the spines and dendrites (Fig. 4I). Among the total, 27 spines showed a positive voltage difference, which we interpreted as likely initiated within the spines (Fig. 4J). Next, we plotted the estimated voltages of this subset of spines and compared them with the voltages measured in the soma using patch clamp recordings [estimated spine voltage = 10.81 ± 4.78 mV, estimated dendrite voltage = 7.41 ± 2.93 mV, and measured cell body = 0.28 ± 0.35 mV (means ± SD); Fig. 4K]. Only a weak correlation was observed between spine depolarization amplitudes or attenuation and distance to the soma (fig. S3D). Considering the same exponential decay function and electrotonic effective length constant described above and the measured distances from spines to the soma (Fig. 4F), we calculated the expected voltage decay according to cable theory. As before, we also found that the measured somatic voltage was significantly lower than the predicted value [expected cell body = 2.72 ± 1.54 mV (means ± SD); Fig. 4K]. Under awake conditions, optogenetically evoked dendritic depolarizations still showed marked attenuation, consistent with our observations under anesthesia (fig. S7E). These results suggest that synaptic activation is attenuated by the dendritic tree to a greater extent than predicted by cable theory. Somatic depolarization appears to occur only when synaptic inputs cooperate, mostly in a synchronized or clustered manner.

## DISCUSSION

To better understand how basal dendrites integrate synaptic inputs in vivo, we imaged voltage changes in spines and dendrites of neocortical pyramidal neurons while simultaneously recording their somatic potentials with whole-cell patch clamp. Across four types of synaptic activity—spontaneous activity, sensory-evoked responses, extracellular electrical stimulation, and presynaptic two-photon optogenetics—we observed two regimes of dendritic transmission. The first regime, driven by synchronous or multiple synaptic sites of activations, led to somatic depolarization. These events often generated dendritic spikes and presumably also reflected the linear summation of spine potentials at various locations within the dendritic tree. In contrast, a second regime was observed during the activation of isolated synaptic inputs, which produced little or no detectable somatic depolarization across the four conditions examined. Our definition of isolated synaptic potentials is operational and based on the absence of detectable coincident activity across imaged compartments and therefore cannot exclude the contribution of distant subthreshold synaptic inputs, which we cannot image. While it is well established that multiple synaptic inputs can drive somatic depolarization, the integration of isolated synaptic events has not been widely studied, particularly in vivo. This gap is partly due to the technical challenges of selectively activating individual dendritic spines and measuring their resulting depolarizations in situ. Our results suggest the existence of a threshold beyond which a considerable portion of spine activity remains undetectable to the soma, becoming effective only through the cooperative action of multiple inputs.

A limitation of our study is the use of anesthesia, which facilitates intracellular recordings in living mice by improving stability and reversibly reducing ongoing activity that might otherwise mask isolated EPSPs (*25*, *34*, *35*, *37*, *49*, *81*). However, anesthesia may alter interneuron activity and, consequently, affect feedback connections (*82*, *83*). It could also influence metabotropic neurotransmission, neuronal input resistance, and synaptic background activity (*23*, *33*, *65*), which would likely affect its integrative properties and dendritic signaling (*84*, *85*). While this could be considered a caveat, our control experiments are consistent with attenuation also present under awake conditions. Although extending these measurements to fully awake animals represents an important future direction, several technical limitations currently prevent a direct replication of the core experiments under these conditions. The principal strength of our approach relies on the combination of targeted dendritic spine voltage imaging with simultaneous somatic whole-cell patch clamp recordings. Achieving stable whole-cell access in awake head-fixed animals remains extremely challenging and would likely preclude obtaining the prolonged dual recordings required for our analyses. Moreover, even if these recordings were technically feasible, the elevated levels of ongoing synaptic and spiking activity characteristic of the awake cortex would make the isolation of individual synaptic events considerably more difficult. While somatic APs could potentially be reduced by hyperpolarizing current injection, the underlying barrage of subthreshold activity would still obscure small spine depolarizations. Resolving individual events under these conditions would likely require averaging across a substantially larger number of trials. However, with current GEVIs and imaging configurations, photobleaching and recording stability impose practical limits on acquisition duration. For these reasons, we restricted the present study to anesthetized conditions, which permit the isolation and direct comparison of synaptic signals across spine, dendrite, and soma. Future improvements in sensor performance and awake whole-cell recording methodologies will be necessary to fully extend these measurements to the awake state. Together, our results are consistent with a model in which only synchronous or clustered activity drives reliable somatic depolarization. During active cortical states, weak inputs can be selectively amplified, particularly in basal dendrites, through subthreshold voltage–dependent mechanisms (*86*), a phenomenon observed under both anesthetized and awake conditions. Whether this amplification reflects dendrite-specific activity coincident with the stimulated synapses, or a more global form of dendritic excitability, remains an open question.

The absence or reduced size of somatic EPSPs in vivo that we document is consistent with previous electrophysiological studies of monosynaptic connections in pyramidal neurons (*87*, *88*). This phenomenon can be partially explained by cable filtering (*89*), which significantly attenuates synaptic potentials. We observed that APs propagate into the dendrite with predictable attenuation, whereas the attenuation of synaptic activity was not well explained by distance. The magnitude of reduction observed in our study therefore suggests that additional mechanisms may also contribute to filtering or blocking synaptic potentials and further limit their propagation to the soma. As mentioned, active background conductances in dendrites (*90*), generated by ongoing activity, could shunt the current injected at the synapse (*91*, *92*). In addition, dendritic inhibition may play a notable role, as γ-aminobutyric acid (GABA)–releasing inputs selectively target the dendritic shafts of pyramidal neurons while largely avoiding the spines (*93*). Dendritic shafts are mostly devoid of excitatory inputs, suggesting that inhibitory inputs in the dendritic shaft may control the propagation of excitatory inputs (*94*, *95*). Furthermore, the stronger activation of inhibitory inputs in vivo could dominate cortical activity due to the widespread connectivity of interneurons (*64*, *96*–*98*). Thus, the primary reason why individual synaptic inputs fail to propagate to the soma could be due to shunting or hyperpolarizing potentials in the dendritic shaft, mediated by inhibitory interneurons.

Regardless of the exact mechanisms underlying the attenuation of synaptic events, our results highlight the importance of coincident synaptic activity in the operation of cortical circuits. We show that activation of multiple synapses can overcome dendritic filtering and generate significant somatic depolarizations. This may arise either from the summation of numerous coactive synapses or from the initiation of local dendritic spikes by clustered excitatory inputs. Although our data do not exclude linear summation as a parsimonious explanation, we also consider nonlinear dendritic integration to be a plausible contributing mechanism. Dendritic electrogenesis has been extensively described in pyramidal neurons in vitro (*5*, *99*–*101*) and may be even more important in vivo, given the strong filtering of synaptic events. Dendritic synaptic clustering by pyramidal cells has been controversial, with some groups demonstrated clustering of functionally similar inputs (*102*–*104*), while others do not (*45*, *49*, *81*). Regardless, pyramidal neurons may be particularly sensitive to synchronous inputs and, as proposed by Abeles, neocortical circuits could be organized to preferentially propagate synchronous activity (*105*). Ensembles of coactive neurons dominate cortical activity (*106*), are causally linked to perceptual and behavioral tasks (*28*, *42*, *104*, *107*–*110*), and are expected to generate synchronous synaptic inputs onto target cells. Thus, pyramidal neurons may be particularly effective at integrating ensemble activity, while single-neuron inputs contribute relatively little to somatic depolarization under these conditions. Moreover, ensemble activation could trigger dendritic electrogenesis as a postsynaptic consequence of synchronous or clustered inputs (*12*, *28*, *42*, *51*, *107*, *108*, *111*, *112*). From this perspective, dendritic spikes may function as cellular detectors of neuronal ensembles, translating circuit events into specific intracellular signals. In this model, dendrites are not merely passive transmitters of EPSPs to the soma but central to the computation of cortical circuits.

## MATERIALS AND METHODS

### DNA construct cloning

All DNA cloning procedures were conducted through polymerase chain reaction fragment ligation using PrimeSTAR GXL DNA polymerase and In-Fusion HD Cloning Plus (Takara Bio). Subsequently, all resulting plasmids were sequenced and purified using NucleoBond Xtra Midi Plus EF (Macherey-Nagel). For the construction of pAAV-CAG-Flex-postASAP5, the PSD95.FingR fragment was obtained from pCAG-PSD95.FingR-eGFP-CCR5TC (Addgene, #46295) and subcloned upstream of the ASAP5 open reading frame (*62*). The resulting fragment was then cloned into the backbone of pAAV-CAG-Flex-GCaMP6s (Addgene, #100835), replacing the GCaMP6s cassette. For the construction of pAAV-hSyn-CreOff-ChRmine, the ChRmine open reading frame was amplified from pAAV-hSyn-ChRmine-mScarlet-Kv2.1 (Addgene, #130995) and subcloned without destination signals into the recipient vector Cre-off-hSyn-CheRiff-CFP (Addgene, #104119), replacing the ChRiff cassette. The pCAG-Cre vector was obtained from Addgene (#13775).

### In utero electroporation

Animal handling and experimentation were conducted in accordance with the guidelines of the US National Institutes of Health and the Columbia University Institutional Animal Care and Use Committee (protocol #AC-AABN3562). Mouse embryos at embryonic day 16.5 were subjected to in utero electroporation as previously described (*61*). Pregnant mice (CD-1, Charles River) were anesthetized with isoflurane [3% (v/v) for induction and 2% (v/v) for surgery] and maintained at 37°C. Presurgery medication included an intraperitoneal injection of carprofen (5 mg/kg), followed by postoperative carprofen doses every 12 hours for 2 days. Uterine horns were exposed by laparotomy, and plasmids at a final concentration of up to 1.0 μg/μl mixed with FastGreen (0.05%, w/v) were injected into the left lateral ventricle of embryos using a glass micropipette. Throughout the procedure, the uterine horn was continuously soaked with warm phosphate-buffered saline. Electroporation was performed using platinum disk electrodes positioned on the lateral side of the embryos’ heads (Nepa Gene, #CUY650P5) and a second electrode positioned at the top of the developing cortex (Nepa Gene, #CUY7003L). Electrical pulses were delivered through the electrodes using an electroporator (Nepa Gene, #NEPA21). Following the procedure, the uterine horn was returned to the abdominal cavity, and the skin was closed with sutures.

### In vivo electrophysiology

In vivo two-photon targeted electrophysiology was conducted following a procedure similar to described previously (*61*). Animals that had undergone electroporation and were between 4 and 8 weeks old (both males and females) were used for this procedure. During the craniotomy and headplate implantation, mice were anesthetized with isoflurane at a concentration of 2% (v/v), which was subsequently reduced to 1 to 1.5% (v/v) during the patch procedure, and maintained at 37°C. A custom-made stainless steel or plastic headplate was affixed to the skull using cyanoacrylate adhesive and dental cement above the left somatosensory area. A craniotomy measuring 1.5 mm by 3 mm and centered in the barrel cortex (−3 mm lateral and −1 mm anteroposterior from bregma) was performed, and the dura mater was removed in the entire area. To ensure optimal imaging quality for dendritic and spine imaging, it was crucial to prevent bleeding or blood clots during the duratomy process. To minimize movement artifacts and facilitate two-photon imaging at the patch clamp recording site, the craniotomy was covered with a custom-made glass measuring 1 mm by 4 mm, leaving a 0.5-mm edge for pipette insertion.

Patch pipettes with a resistance of 10 to 12 MΩ were fabricated using borosilicate glass (1.5 mm in outer diameter and 0.86 mm in inner diameter; Sutter Instruments) and pulled with a horizontal puller (Sutter P-97). For whole-cell recordings, pipettes were filled with an internal solution containing 120 mM K-gluconate, 3 mM KCl, 4 mM NaCl, 4 mM Mg–adenosine 5′-triphosphate, 0.3 mM Na–guanosine 5′-triphosphate, 20 mM Hepes, 14 mM tris-phosphocreatine, and 0.01 mM Alexa Fluor 594/488, adjusted to pH 7.3 and 290 mOsm. Positive pressure (300 to 400 mbar) was applied to the patch pipettes until they were inserted into the brain to a depth of 0.1 mm. Subsequently, the pressure was reduced to 50 to 100 mbar to navigate in layer 2/3. Upon reaching the targeted cell, the pressure was further decreased to 10 to 20 mbar for cell approach and gigaseal formation. Once the whole-cell configuration was established with access resistance in the range of 50 to 100 MΩ, recordings were conducted in current-clamp mode with appropriate capacitance compensation and no current injection for membrane potential maintenance. All electrophysiological data were amplified with a MultiClamp700B (Molecular Devices) and acquired at 10 kHz using Prairie View 5.5 software.

### Electrophysiology analysis

Electrophysiological data were analyzed according to the following workflow:

1) Preprocessing of voltage signals: To remove power line interference at 60 Hz from the voltage signal, a narrow-band, second-order notch digital filter was applied to all recordings using zero-phase filtering to prevent phase distortion. In addition, the signal was smoothed using a 10-point moving average.

2) RMP epochs: The RMP was defined as the mean voltage calculated over a stable, quiescent baseline period of at least 100-ms duration (trial average segments), and deviations from this value were quantified as transient changes relative to this mean baseline. After generating a histogram of all membrane potential values from the whole-cell recording, the RMP was defined as the mean of the Gaussian curve that best fit the data in the lower voltage range (i.e., excluding putative subthreshold and suprathreshold events located in the right tail of the curve). The SD of this Gaussian was then used to define the SNR of the voltage events.

3) Detection of subthreshold events: A depolarization was considered a subthreshold event if the RMP deviated by at least 2 mV above the RMP for a minimum duration of 10 ms. No maximum duration was set for these events. For all analyses, trials containing somatic APs detected in patch clamp recordings were excluded, and spine-soma comparisons were restricted to subthreshold conditions.

4) Detection of APs: Any event that crossed a −40-mV threshold was classified as an AP, with individual events defined as those separated by at least 10 ms. In cases where AP trains were detected, a 100-ms interevent interval was required to distinguish individual events.

### Natural CSF injections

Using the same surgical procedures employed for in vivo electrophysiology, injections of TTX (1 μM) dissolved in natural CSF were administered in the barrel cortex (*72*). Natural CSF was extracted by injecting and aspirating fluid from the cisterna magna of mouse littermates not used for in vivo patch experiments. Metal needles and glass syringes (34 g, 10 μl; Hamilton), coupled with a micropump system (Micro 4, World Precision Instruments), were lowered to ∼150 to 200 μm below the pia. Approximately 2 μl of the solution was injected over a period of 10 min. Imaging of dendrites and spines was conducted within a radius of 1 mm around the needle tip. The efficacy of solution diffusion was monitored by injection with FastGreen (0.05%, w/v).

### Anesthesia protocol

Properly calibrated tabletop laboratory animal anesthesia system (VetEquip) was used for all experiments, with airflow set to 2 liters/min. During procedures for establishing whole-cell recordings and during spontaneous recordings, isoflurane anesthesia was maintained at 1.0 to 1.5%. This range was defined as light anesthesia, as subthreshold/suprathreshold activity was observed at a frequency of ∼1 event/s. During sensory-evoked, electrical, or optogenetic recordings, the isoflurane concentration was increased until the subthreshold/suprathreshold activity frequency was reduced to 0.1 to 0.3 Hz, with a maximum concentration of 3% (v/v) applied to avoid respiratory depression.

### In vivo two-photon imaging

All in vivo experiments were performed in a custom-made two-photon microscope (adapted from Prairie model, Bruker), equipped with a 25×/1.05–numerical aperture (NA) water immersion objective (Olympus) and a tunable Ti-sapphire laser (Mai Tai eHP DS). Measurements of the experimental point spread function of the microscope indicated full width at half maximum for lateral and axial resolution of 0.8 and 3.6 μm, respectively. For voltage or calcium imaging, combined with dual-color imaging, neurons were imaged with the laser tuned to 920 nm, and signals were collected using filters of 510/20 nm for postASAP5/GCaMP8 and 605/15 nm for red-shifted fluorophores. Imaging experiments were conducted in layer 2/3 of the left somatosensory cortex (∼0.1 to 0.2 mm from the pia). The voltage sensor was imaged at a resolution of 256 pixels by 40 pixels with 8× to 12× zoom at 350 Hz (pixel size at 1×, 2 μm by 2 μm). The imaging laser power at 920 nm was measured at the end of the objective, and for regular imaging, 80 to 120 mW was used for all experiments.

### Sensory stimulation

Whisker stimuli were delivered to all contralateral vibrissae using a 10-ms air puff at 3-bar pressure (PICOSPRITZER III, Parker) through a 1-mm round plastic tip positioned 1 cm from the vibrissae. Before starting the experiment, it was verified that the stimulus reliably triggered movement in all vibrissae. Stimuli were applied during the imaging of dendritic segments over 10 trials, with 3-s intervals between each trial. The stimulus window, during which subthreshold or suprathreshold activity was observed, was defined as the 1-s period following the air puff. For comparison purposes, the baseline period was defined as the entire recording period excluding the stimulus window.

### Extracellular electrode stimulation

Immediately after the in vivo electrophysiology surgery, the animal was transferred to a two-photon microscope rig, and a concentric bipolar electrode with a diameter of 200/50 µm (outer/inner pole; FHC, #30215) was positioned 1 mm away from the site where the patch pipette entered the somatosensory cortex. Stimulation was conducted using a stimulus isolator (World Precision Instruments, A365) in bipolar mode. Pulses of 1-ms duration were generated by a Master-8 pulse generator (AMPI) to the isolator. Microstimulation amplitudes were individually adjusted within the 2- to 10-mA range to reliably evoke detectable subthreshold voltage responses at the soma and/or spines, while avoiding suprathreshold responses or widespread network activation.

### Viral injections

Mice were anesthetized with 2% isoflurane and placed in a stereotaxic frame atop a heating pad maintained at 37°C. Enrofloxacin (5 mg/kg) and carprofen (5 mg/kg) were injected intraperitoneal and lidocaine (2 mg/kg) subcutaneously in the site of the skin incision over the midline of the scalp. A 0.5-mm hole was drilled in the skull over the anterior left portion of the somatosensory cortex. Viral preparations pAAV-axonal-jRexGECO1a or pAAV-ChRmine-mScarlet (both packaged in adeno-associated virus serotype 5, AAV5) were injected into layer 2/3 of the primary somatosensory barrel cortex (3 mm lateral, 0 mm posterior, and 1.6 mm down from the bregma) to target intracortical axons of pyramidal cells. The viral prep (150 nl) was injected at a rate of 50 nl/min, and a 3-min wait period before needle withdrawal.

### Two-photon optogenetic stimulation

We used voltage imaging to monitor the activity of dendrites and spines upon presynaptic photostimulation. Two-photon imaging and optogenetic stimulation were conducted using two femtosecond-pulsed lasers integrated into the microscope setup. The imaging laser was set to 920 nm, while the photostimulation laser operated at 1064 nm (Fianium; pulse duration, 400 fs; maximum pulse energy, 50 nJ; NKT Photonics) to excite the red-shifted opsin ChRmine, which responds to longer wavelengths. Laser power was measured after the objective and calibrated according to the Pockels cell voltage command. Before each session of imaging and photostimulation, precise calibration alignment of the stimulation laser was performed.

The two laser beams directed onto the sample were independently controlled by separate sets of galvanometric scanning mirrors. The typical image field of view was ∼100 μm by 10 μm with a 25×/1.05 NA water immersion objective. Circular ROIs for photostimulation (automatically drawn by Prairie software) were selected outside the dendritic shaft (10 μm in diameter), guided by the fluorescence displayed online by the voltage sensor, using spiral stimulation (10 to 15 revolutions). Time-locked photostimulation was carried out with intervals of 1 s for each trial (30 trials). The stimulation duration was set 10 to 100 ms. Potential optical cross-talk between stimulation light and voltage imaging signals was excluded on the basis of polarity: Depolarization produces negative Δ*F*/*F* with postASAP5, whereas stimulation artifacts would appear as positive signals.

### Awake two-photon imaging

After headplate implantation and postoperative recovery, mice were habituated over multiple days to head fixation on the recording apparatus. On the day of the experiment, animals were briefly anesthetized with isoflurane (∼2%) for craniotomy and glass window implantation, as previously described, and then head-fixed on the recording setup. Anesthesia was subsequently discontinued, and mice were allowed to recover fully to an awake, responsive state before recordings began. During recordings, mice remained head-fixed on the behavioral platform. The brain surface was covered with modified Dulbecco’s phosphate-buffered saline (Sigma-Aldrich), and recordings were initiated after a 20- to 30-min recovery period. Data were acquired only from mice that, after habituation, displayed little to no movement on the platform, and imaging was restricted to periods of minimal movement to minimize motion-related artifacts.

Motion correction was performed using Suite2p (*113*) with custom parameterization. For each recording, a reference image was computed by calculating the Pearson correlation between each frame and the global mean image, selecting the 500 most highly correlated frames, and averaging them to generate a stable reference template. Rigid registration was then applied to estimate global *x*-*y* displacements of each frame relative to this reference, using a two-step registration procedure. Temporal and spatial Gaussian smoothing (σ = 1) were applied during registration to stabilize displacement estimation. Motion-corrected image batches were saved as registered Tagged Image File Format (TIFF) stacks and subsequently concatenated into a single time-ordered memory-mapped array to preserve acquisition order across batches. Rigid displacement vectors were retained for downstream quality control, including estimation of motion magnitude and drift over time.

Following rigid motion correction, image sequences were denoised using SUPPORT (statistically unbiased prediction using spatiotemporal information in imaging data) (*114*), a self-supervised deep-learning framework for functional imaging data. Denoising was performed by inference using a provided pretrained model with a blind-spot size of 1, without additional training on the present dataset. The motion-corrected movies were divided into overlapping three-dimensional patches, each spanning 61 consecutive frames and 32 pixels by 64 pixels in space, and the denoised patches were subsequently reassembled to reconstruct the full image sequence. Patches were extracted with sliding steps of one frame in time and 16 and 32 pixels in the two spatial dimensions. For each 61-frame window, the model generated a denoised estimate of the central frame using the surrounding frames as temporal context. The denoised outputs were then restored to the original intensity scale and saved as reconstructed image stacks for downstream analyses.

### Imaging analysis

The image analysis workflow consisted of the following steps:

1) Times series preprocessing pipeline: Image analyses were conducted using routines written in MATLAB, Python, and ImageJ. Rigid body movement correction was performed using TurboReg. The template for movement correction was generated by averaging the first 1000 frames of the time series. Motion correction was applied primarily to compensate for heartbeat and respiration related lateral displacements in the *x*-*y* plane, whereas residual axial (*z*) motion was minimized by surgical stabilization. In cases where shivering or strong motion along the *x*-*y* axis was observed, the corresponding periods were either discarded, or the entire movie was rejected from the analysis. No blood autofluorescence or other artifacts were detected in our imaging recordings. Then, motion-corrected movies were denoised using spatially localized penalized matrix decomposition. This method exploits two properties of functional imaging data: (i) Fluorescent signal sources are spatially local, and (ii) signal exhibits temporal and spatial structure, while noise does not. By conducting principal components analysis in patches of the image (set to 20 pixels by 20 pixels), the algorithm calculates local filters to discard uncorrelated pixels throughout the entire movie, resulting in a denoised video. Processed videos were subjected to a Kalman filter with an estimated noise variance of 5% and an 80% gain. Because of nonuniform bleaching decay across the field of view, each pixel in the denoised movie underwent correction for photobleaching using a spline function with a time window of 5 s. The resulting curve was then subtracted point by point from the original raw pixel values. Inverted voltage signal peaks were computed as a change in fluorescence: (*F* − *F*_i_)/*F*, where *F*_i_ denotes the fluorescence intensity at any frame and *F* denotes the spline fitting of the signal.

2) Fluorescent signal extraction: ROIs outlining spines, dendrites, and the soma were manually selected. Somatic ROIs were drawn to match the sizes of the spines and dendrites, and a background ROI was chosen as close as possible to the targeted compartment with the same number of pixels. The number of pixels varied according to the size of the structure, generally ranging from 20 to 60 (38.8 ± 13.7; means ± SD). The fluorescent signal for each ROI was calculated as the average intensity of all selected pixels. In the subsequent analysis, data were analyzed at the level of individual spines and corresponding dendritic segments. For each recorded spine, all detected events (with a minimum of three events) under different conditions, APs, RMP, subthreshold, and pre- and poststimulus (sensory-evoked, electrical, or optogenetic), were averaged to calculate Δ*F*/*F*.

3) SNR calculations: SNR of detected events was estimated for both individual trials and trial-averaged signals. For individual trials, the SNR was calculated as the ratio of the peak fluorescent change to the SD of the entire recording (also characterized as *z*-score). For trial-averaged signals, the SNR was calculated as the ratio of the peak fluorescent change following the time-locked stimulus to the SD of the trace preceding the stimulus. In addition, the SNR was calculated as the peak of Δ*F*/*F* over the SD of the mean ROI located in the background.

4) Calibration of fluorescence response to voltage: For in vivo electrical recordings coupled with two-photon imaging, ROIs selected on the soma were matched in size to those selected on spines and dendrites. Electrical subthreshold events corresponding to a 4-mV change from the RMP were automatically detected as individual events within a 100-ms window, with no consecutive events occurring within 200 ms. Once identified, the RMP was calculated as the average membrane potential during the 100-ms period preceding the event, and the peak was defined as the maximum voltage change within the 100-ms window of the event. The corresponding peak in fluorescence change was detected within the same time frame. From this correlation analysis of 435 subthreshold events, the average SNR was 2.67 ± 1.32 (means ± SD), with 66.9% of events exceeding the SNR > 2 detection threshold (false-negative rate = 33.1%; fig. S2A). To establish a baseline reference, we applied the same detection parameters to the basal period, that is, the RMP epochs preceding the onset of subthreshold events. Under this condition, putative signals had an average SNR of 1.08 ± 0.71 (means ± SD), with only 10.3% of events exceeding the SNR > 2 threshold (false-positive rate; fig. S2A). All subthreshold events were then binarized into 1-mV increments to calculate the average fluorescence change, and the correlation/linear fitting between fluorescence change and electrical voltage change was subsequently calculated. All Δ*F*/*F* – *V* transformations were restricted to subthreshold events within the linear range of ASAP5; suprathreshold signals were excluded from quantitative analysis due to sensor nonlinearity.

5) Measurement of spine-to-soma distances: Relative *x*, *y*, and *z* position data were acquired during imaging recordings. The soma was set as the origin (zeroed), and the distance to each spine was calculated using the center of mass of the ROIs drawn for spines. Euclidean distance was then calculated to determine the relative distance from the soma to each spine. These Euclidean estimates were corroborated against the actual dendritic morphology, following the course of basal dendrites, and corrected accordingly in cases where non-Euclidean paths were observed.

6) AP propagation into dendrites and spines analysis: After detecting individual APs in the electrical recordings, the corresponding time-locked fluorescence change in dendritic and spine ROIs was analyzed within a 30-ms window following each somatic AP. To calculate the probability of activation, spine signals during APs were considered reliable if they exhibited an SNR of >2. Fluorescence change of spines was then averaged across all APs recorded to determine the overall response.

7) Calculation of spine-specific or spine + dendrite events: According to mathematical modeling of dendritic spines, the voltage transfer from dendrites to spines should be close to 99% (*115*) because spines are not isolated from voltages in the parent dendrite. On this basis, we assume that any variations observed in our experiments when comparing AP-evoked fluorescent signals in dendrites and spines could be attributed to concurrent signals or imaging noise. To assess this, we plotted the difference in fluorescence change between spines and dendrites during APs, which resulted in a normal distribution. This suggests that dendritic events transferred to spines should follow a linear relationship with no decay, and any deviation from this could be modeled as differences between spine-dendrite compartments. We calculated the fluorescence change (or estimated voltage) differences between spines and dendrites during both electrical and optogenetic activation. By plotting these differences, we confirmed a normal distribution centered around zero. To establish a threshold, we set it at 1 SD of this distribution. Events within this range were classified as spine + dendrite events, while those in the right-tail of the distribution were classified as spine specific, indicating that spine activation exceeded the corresponding dendritic segment activation.

### Expected somatic voltage calculations

Derived from dendritic modeling as cylindrical core conductors, passive cable theory describes dendrites in one dimension using the passive cable equation. A ball-and-stick model, which simplifies neuronal morphology by coupling a cylindrical dendritic compartment with a spherical soma, captures essential voltage transfer properties (*116*). One key biophysical parameter of the dendritic tree is the effective electrotonic length constant (λ). On the basis of cable theory and morphometric estimations (*117*), we assumed a model of arbitrary passive dendritic branching, without incorporating membrane conductances, as these would require a dynamic model. In this framework, the voltage at any point along the dendrite (*V*_dendrite_) decays exponentially with distance (*l*) to the voltage in the soma (*V*_soma_) according to the equation

$$V_{\text{soma}}=V_{\text{dendrite}}e^{-l/\lambda }$$

This equation quantifies the efficacy of voltage transfer in terms of an e-fold drop. To estimate the expected voltage decay at the soma, we applied this passive cable model based on prior studies (*117*). In layer 5 pyramidal neurons, basal dendrites have been estimated to have a length constant of λ = 50 μm, indicating significant voltage attenuation, similar to that observed in apical dendrites (*12*). Other studies have reported higher values, such as λ = 90 μm for basal dendrites (*62*) and λ = 188 μm (*32*) or λ = 273 μm (*91*) for apical dendrites in the neocortex. In our calculations, we used λ = 50 μm as a reference to estimate the expected somatic voltage attenuation based on the equation above.

### Statistical analysis

Statistical analyses were performed with Prism 10 software (GraphPad) or MATLAB built-in functions. *P* > 0.05 was considered as not statistically significant (ns). To report *P* values, we used *P ≤ 0.05, **P ≤ 0.01, ***P ≤ 0.001, and ****P ≤ 0.0001. In general, normality of the data groups was confirmed by Shapiro-Wilk and/or Kolmogorov-Smirnov tests. Detailed statistical methods used in the experiments are provided in the figure captions or the Results section. In graphs, each data point is represented individually, with bars and lines indicating the mean. Box and whiskers depicted median (line), 25th to 75th percentiles (box), and range (whiskers), and mean represented as a “+.”
