# Supplementary material for: Highly attenuated dendritic propagation of isolated synaptic potentials in vivo
Source: Sci Adv. 2026 Aug 14;12(33):eadz4123. doi: 10.1126/sciadv.adz4123 (PMC13475487; doi:10.1126/sciadv.adz4123)
Supplement: Supplementary file 1 [file sciadv.adz4123_sm.pdf]

Science Advances
AAAS

Supplementary Materials for

Highly attenuated dendritic propagation of isolated synaptic potentials in vivo

Victor Hugo Cornejo *et al.*

Corresponding author: Victor Hugo Cornejo, victor.cornejo@uc.cl; Rafael Yuste, rmy5@columbia.edu

Sci. Adv. **12**, eadz4123 (2026)
DOI: 10.1126/sciadv.adz4123

This PDF file includes:

Figs. S1 to S7

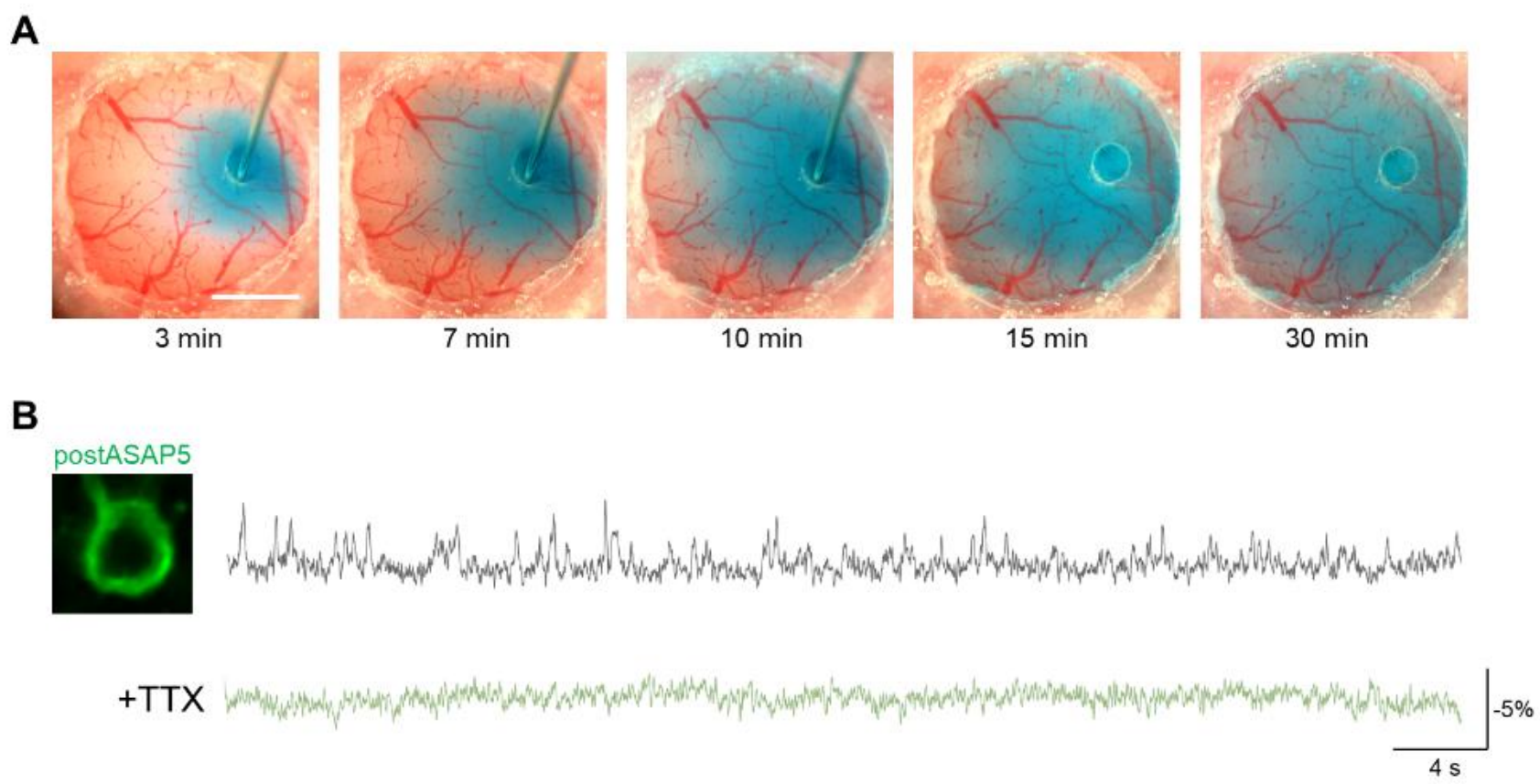

Figure S1. Intravital injection of TTX and voltage imaging in the cell body.
(A) Cranial window in the mouse somatosensory cortex with a 3 mm glass implant containing an off-centered hole (scale bar = 1 mm). Example of natural CSF injection labeled with FastGreen dye, showing the diffusion of the dye over time. **(B)** Voltage imaging of a cell body, displaying traces before TTX application (gray) and 30 minutes after injection (light green).

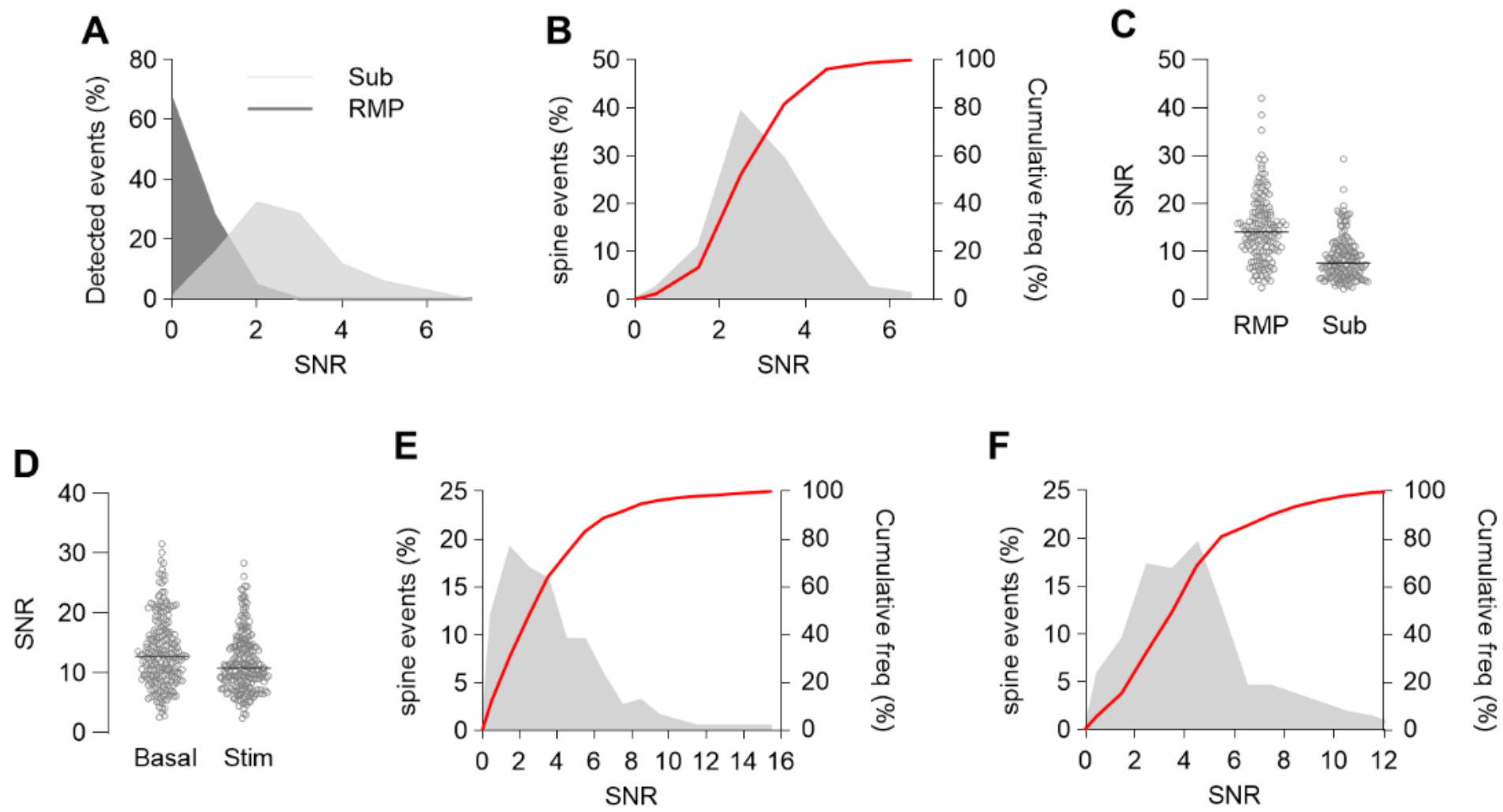

Figure S2. Distributions of SNR values for voltage imaging signals across experimental conditions.

For all calculations, SNR was computed as the ratio of the peak fluorescence amplitude to the standard deviation of the baseline signal measured in the 100 ms preceding event detection. **(A)** Histogram of SNR for somatic voltage events. The distribution of SNR values is shown for 435 subthreshold events (Sub), with an average SNR of 2.67 ± 1.32 [mean ± SD]; 66.9% of events exceeded the SNR > 2 detection threshold. For comparison, the same detection parameters were applied to basal RMP epochs preceding subthreshold events, yielding an average SNR of 1.08 ± 0.71 [mean ± SD], with 10.3% of events crossing the threshold. **(B)** After APs detection in the soma, the percentage and cumulative distribution of SNR values are shown for each spine depolarization event. **(C)** SNR calculated after trial averaging of detected spine events, classified according to whether they occurred during subthreshold epochs (Sub) or at RMP in spontaneous recordings. **(D)** SNR calculated after sensory stimulation, with spine events classified as occurring within 1 s following whisker stimulation (Stim) compared to the total recording period (basal). **(E)** SNR distributions after averaging of electrically evoked spine events, plotted as in (B). **(F)** Same analysis as in (E), but for optogenetically evoked spine events.

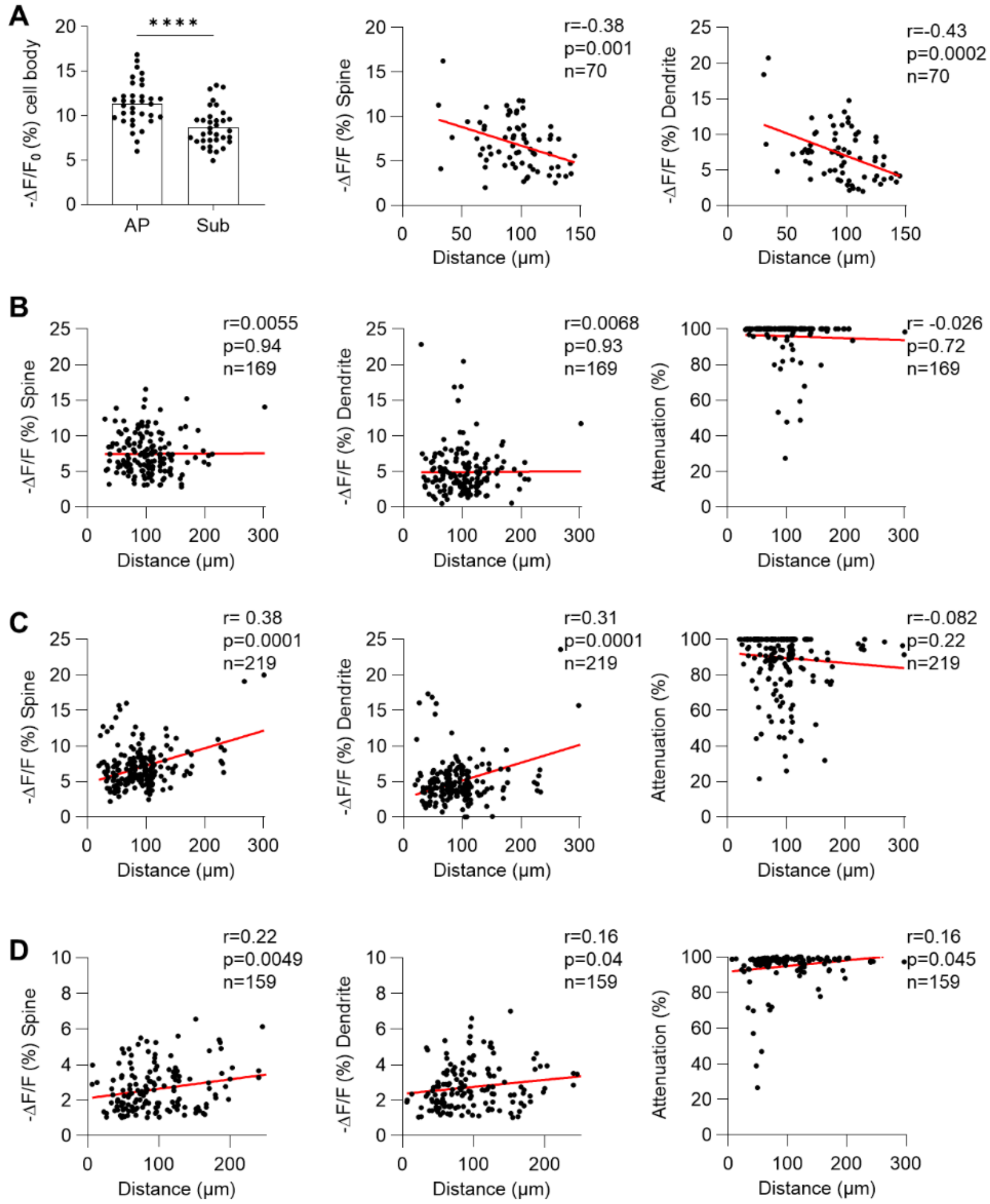

Figure S3. Correlation of APs and synaptic signal amplitudes with spine-to-soma distance. **(A)** On the left, quantification of peak amplitudes for APs and subthreshold events recorded at the soma. AP = 11.35 ± 2.50 −ΔF/F%, Sub = 8.67 ± 2.15 −ΔF/F% [mean ± SD], Mann-Whitney test, $p < 0.0001$. On the center and right, fluorescence amplitude for each spine and adjacent dendritic shafts during back-propagating APs was correlated with its distance from the soma. **(B)** Scatter plots showing correlations between spine fluorescence amplitude, dendritic shaft amplitude, and spine-to-soma distance during RMP. The third plot shows attenuation, calculated as the percentage difference between dendritic voltage estimated from GEVI signals and the time-locked somatic voltage measured by patch clamp. **(C)** Equivalent analysis for sensory-evoked activity, showing spine and dendritic event amplitudes correlated with spine-to-soma distance and corresponding attenuation. **(D)** Equivalent analysis for optogenetically evoked activity, showing spine and dendritic event amplitudes versus spine-to-soma distance and attenuation.

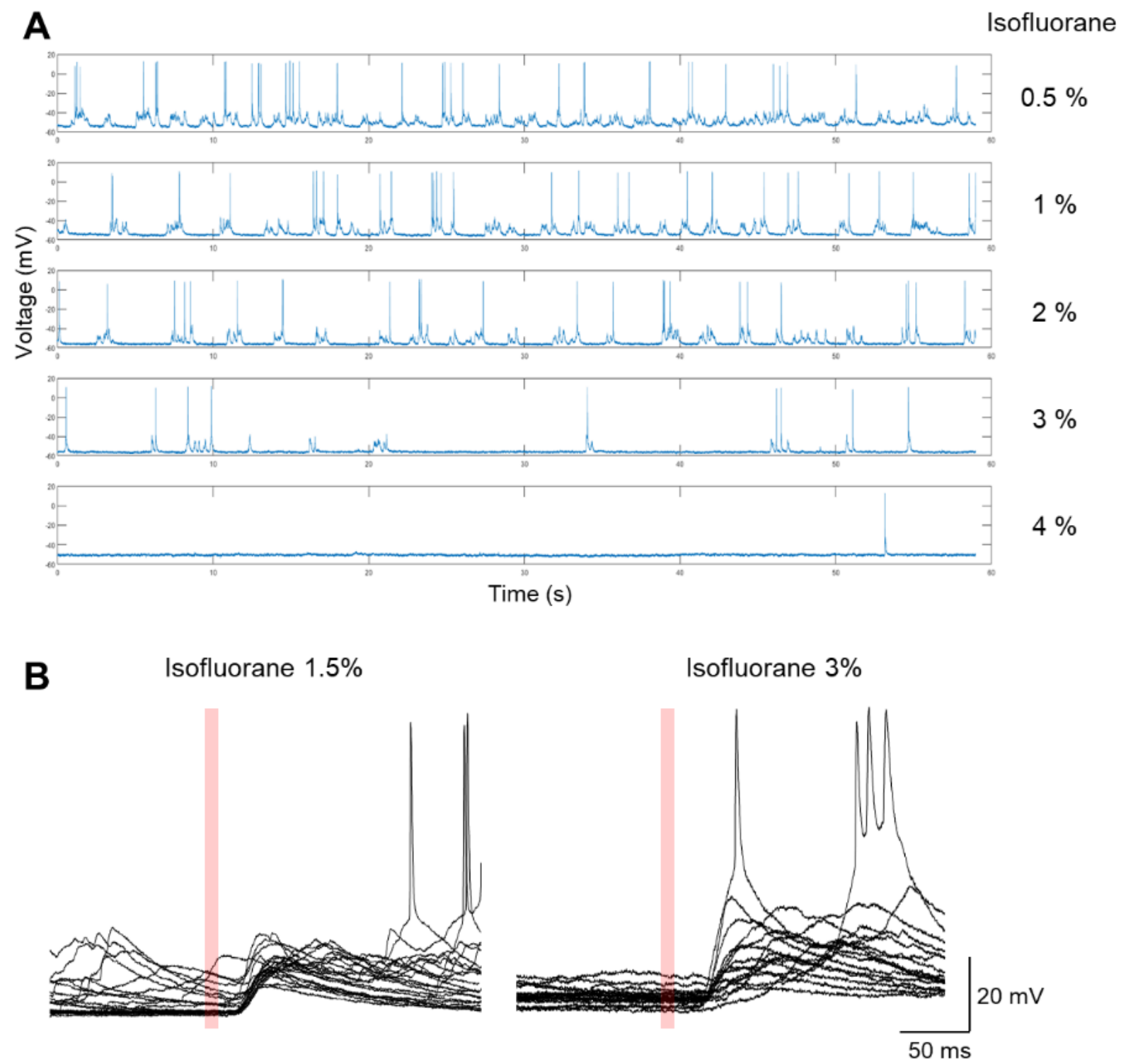

Figure S4. Neuronal activity under different anesthesia planes with varying isoflurane levels. **(A)** Example of an *in vivo* whole-cell patch clamp recording from a neuron in the somatosensory cortex during spontaneous activity. Animals were anesthetized with different concentrations of isoflurane. **(B)** Trial-averaged signals following whisker stimulation at two different anesthetic concentrations (red line).

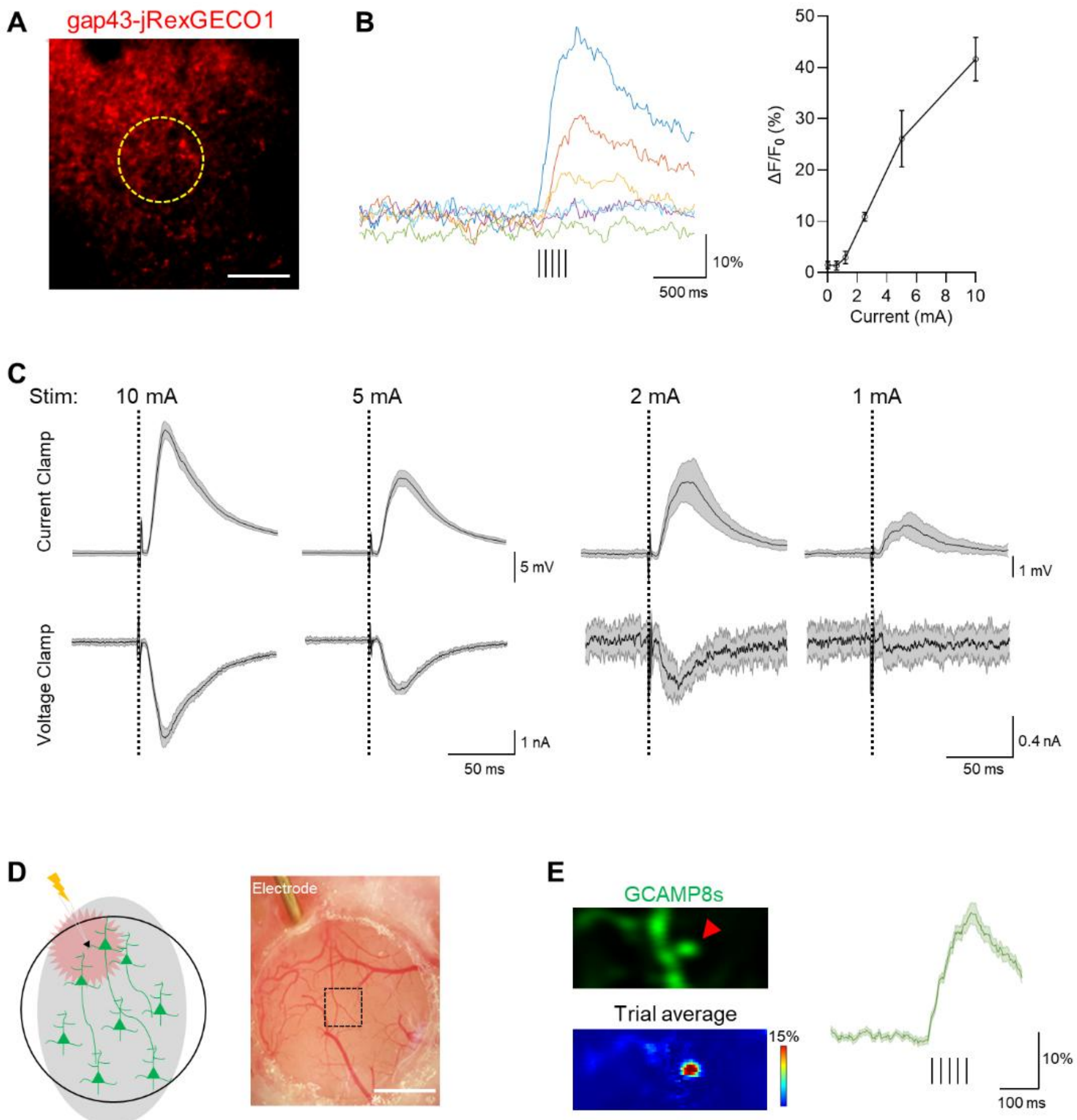

Figure S5. Imaging and electrophysiological responses after electrical stimulation.
(A) Axonal calcium imaging using gap43-jRexGECO1a in remotely stimulated axons. Viral expression of gap43-jRexGECO1a, which preferentially localizes to axons (scale bar = 120 μm). The stimulation electrode is positioned 1 mm away from the imaging site. **(B)** Imaging of a population of axons following a train of brief stimuli at varying current intensities. Single-trial traces are shown in different colors. Right: Relationship between stimulus intensity and axonal calcium imaging responses. **(C)** Calibration of electrical neuropil stimulation and postsynaptic responses in whole-cell patch clamp. Example of an *in vivo* whole-cell patch clamp recording of a neuron in the somatosensory cortex during electrical stimulation. A bipolar electrode was positioned approximately 1 mm from the postsynaptic cell within the somatosensory area. The example neuron was recorded in either voltage-clamp or current-clamp mode under different stimulation intensities. The data shown represent the full range of stimulation intensities tested; for all experiments, amplitudes were selected from the lower-middle range (2-10 mA) to elicit reproducible subthreshold responses while avoiding suprathreshold activation. **(D)** Experimental strategy. Expression of CAG-GCaMP8s-Flex with CAG-Cre for sparse labeling of neurons. A bipolar electrode was inserted into layer 2/3 of the brain for simultaneous presynaptic stimulation and postsynaptic imaging. The gray shaded area represents the mouse primary somatosensory cortex, and the position of the 3 mm cover glass is outlined in black. Right: Example of intravital

electrode insertion and the imaging site (box, dashed line) located approximately 1 mm away from the electrode (scale bar = 1 mm). **(E)** In a neuron expressing GCaMP8, trains of remote electrode stimulation were applied, and calcium signals were recorded in spines and dendrites. In this example, calcium imaging of a spine following a train of short stimuli (10 trials, 5 pulses, 40 Hz) shows spine-specific activation. Left: Z-projection of the dendritic segment and trial-averaged image frames after stimulation. Right: Averaged trace (green) of the spine ROI marked by the red arrowhead.

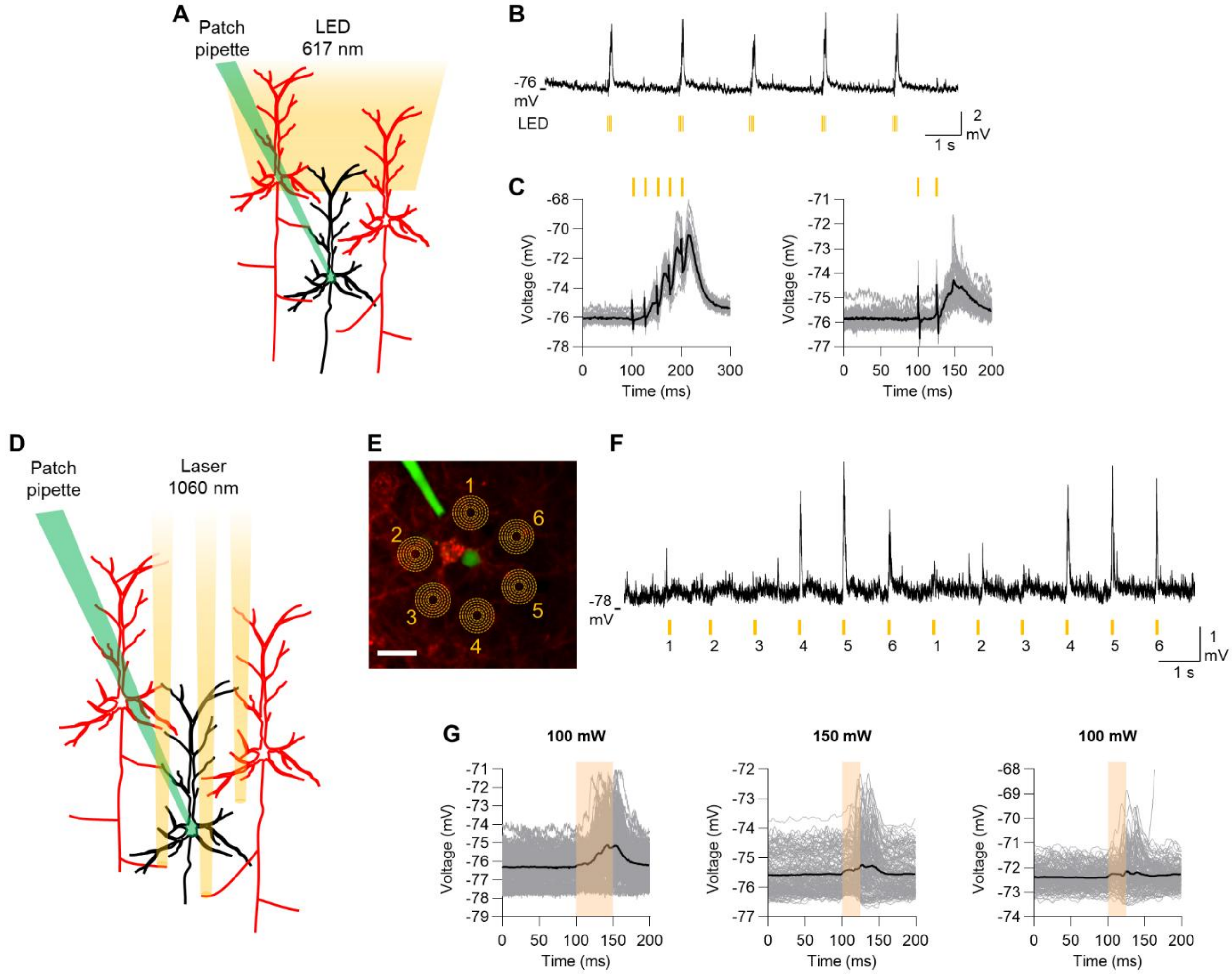

Figure S6. Presynaptic optogenetic stimulation of neurons expressing ChRmine.
(A) Experimental strategy. LED illumination was applied to the brain surface through an optical cannula. Targeted *in vivo* patch clamp recordings were performed on a neuron expressing postASAP5. **(B)** Example trace of whole-cell recording from a neuron expressing postASAP5, showing postsynaptic responses following light stimulation trains with LED (1 ms × 5 pulses, 40 Hz). **(C)** Trial average of 30 trains with varying pulse numbers from 5 to 2 per trial. Gray traces represent individual trials, while the black trace shows the average of all trials. Light orange lines indicate the timing of light pulses. **(D)** Experimental strategy. A randomly selected neuron lacking ChRmine-mScarlet expression was recorded using shadow patch clamp, while perisomatic stimulation was applied with a two-photon laser. **(E)** Example of a neuron filled with Alexa Fluor 488 dye using the patch pipette. Two-photon laser stimulation was conducted using a spiral raster scan. Circular stimulation ROIs, each 20 μm in diameter, were positioned around the patched neuron (scale bar = 25 μm). Sequential stimulation of 6 ROIs was performed over 27 rounds. **(F)** Example traces of whole-cell recordings from the neuron stimulated at perisomatic ROIs (numbered in E; 50 ms, 100 mW). **(G)** Trial average of 162 stimulations (all ROIs) with varying pulse durations (50 ms to 20 ms) and laser power. Gray traces show individual trials, while the black trace represents the average of all trials. Light orange shaded areas mark the timing of light pulses.

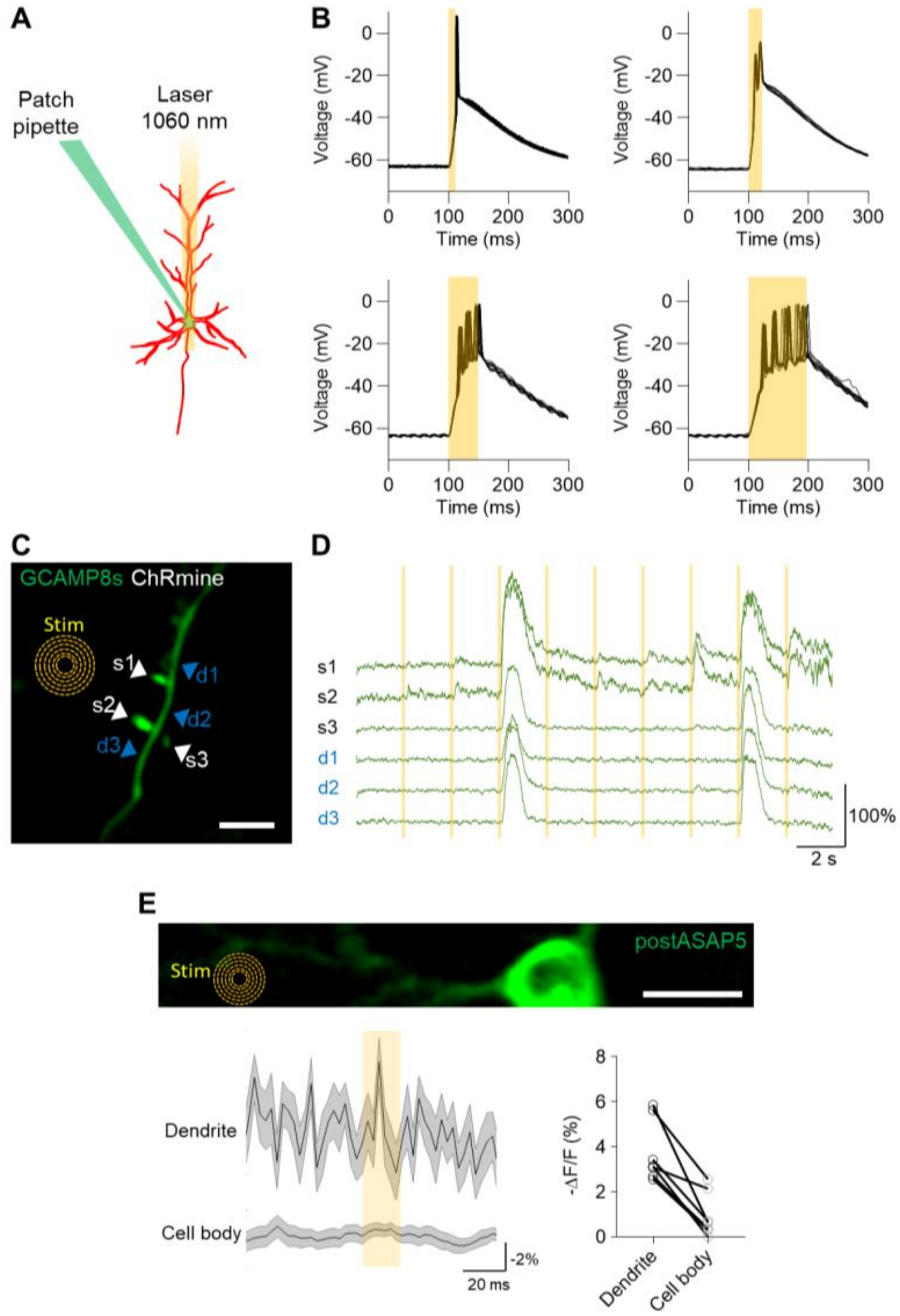

Figure S7. Precise triggering of action potentials with optogenetics and local postsynaptic responses.

(A) Experimental setup. Neurons expressing ChRmine-mScarlet were subjected to *in vivo* whole-cell patch clamp in current-clamp mode, followed by two-photon stimulation at the axonal shaft. The axonal shaft was traced after dendrite mapping and identified as morphologically thinner than dendrites. **(B)** Whole-cell recordings showing responses to stimulation with the same laser power (150 mW) but varying durations 10, 20, 50 and 100 ms, under simultaneous imaging laser illumination. **(C)** Targeted imaging using the expression of CAG-GCaMP8s-flex with CAG-Cre and ChRmine-CreOff (scale bar = 5 µm). Stimulation trials were applied at the same location near the dendrite expressing GCaMP8s (light orange spirals). **(D)** Calcium imaging traces (green) from different ROIs drawn on spines (s) and dendrites (d) following 50 ms of stimulation. **(E)** Top, representative layer 2/3 pyramidal neuron expressing postASAP5 imaged in an awake mouse (scale bar = 10 µm). A two-photon stimulation ROI was positioned adjacent to the dendritic segment (distance dendrite-soma = 35.86 ± 21.01 µm [mean ± SD]). Voltage imaging signals were acquired independently from the dendrite and the soma in consecutive recordings, while

maintaining the same photostimulation site. Left, representative voltage imaging traces recorded from the dendrite and the soma following local optogenetic stimulation. Right, quantification of fluorescence change ($-\Delta F/F\%$) measured in dendritic and somatic recordings, showing stronger local responses in dendrites and attenuated signals at the soma. Data are from n = 7 dendritic segments, 3 animals.
